# Engineering an anti-HER2 biparatopic antibody with a multimodal mechanism of action

**DOI:** 10.1038/s41467-021-23948-6

**Published:** 2021-06-18

**Authors:** Florian Kast, Martin Schwill, Jakob C. Stüber, Svende Pfundstein, Gabriela Nagy-Davidescu, Josep M. Monné Rodríguez, Frauke Seehusen, Christian P. Richter, Annemarie Honegger, Karen Patricia Hartmann, Thomas G. Weber, Felix Kroener, Patrick Ernst, Jacob Piehler, Andreas Plückthun

**Affiliations:** 1 Department of Biochemistry, University of Zurich, Zurich, Switzerland; 2 Zurich Integrative Rodent Physiology (ZIRP), University of Zurich, Zurich, Switzerland; 3 Laboratory for Animal Model Pathology, Institute of Veterinary Pathology, Vetsuisse Faculty, University of Zurich, Zurich, Switzerland; 4 Department of Biology/Chemistry and Center for Cellular Nanoanalytics, Osnabrück University, Osnabrück, Germany; 5 Dynamic Biosensors GmbH, Planegg, Germany; 6 Present Address: TOLREMO therapeutics AG, Muttenz, Switzerland; 7 Present Address: Roche Innovation Center Munich, Penzberg, Germany; 8 Present Address: Dean’s Office and Coordination Office of the Academic Medicine Zurich, University of Zurich, Zurich, Switzerland

**Keywords:** Immunochemistry, Oncogene proteins, X-ray crystallography, Microarray analysis, Protein design

## Abstract

The receptor tyrosine kinase HER2 acts as oncogenic driver in numerous cancers. Usually, the gene is amplified, resulting in receptor overexpression, massively increased signaling and unchecked proliferation. However, tumors become frequently addicted to oncogenes and hence are druggable by targeted interventions. Here, we design an anti-HER2 biparatopic and tetravalent IgG fusion with a multimodal mechanism of action. The molecule first induces HER2 clustering into inactive complexes, evidenced by reduced mobility of surface HER2. However, in contrast to our earlier binders based on DARPins, clusters of HER2 are thereafter robustly internalized and quantitatively degraded. This multimodal mechanism of action is found only in few of the tetravalent constructs investigated, which must target specific epitopes on HER2 in a defined geometric arrangement. The inhibitory effect of our antibody as single agent surpasses the combination of trastuzumab and pertuzumab as well as its parental mAbs in vitro and it is effective in a xenograft model.

## Abstract (web summary)

HER2 acts an oncogenic driver in numerous cancers. Here, the authors design an anti-HER2 biparatopic and tetravalent IgG fusion with inhibitory effects in a xenograft model.

## Introduction

In our aging society, cancer is among the most prevalent diseases. For the onset and development of human tumors, explicit hallmarks were recognized as critical^1^. The hallmark of sustained proliferative signaling is often caused by dysregulated receptor tyrosine kinases (RTKs) acting as tumor drivers^2^. One such driver gene is *ERBB2*, coding for HER2, an orphan member of the epidermal growth factor (EGF) receptor family. Early on, tumor drivers were identified as desirable choices for targeted therapy. The breakthrough discoveries around monoclonal antibody (mAb) technology and engineering enabled specific and actionable targeting of cell surface molecules. Today, a plethora of mAbs is in clinical use or under investigation in clinical trials and preclinical studies^3^.

For HER2-targeted antibody-based therapy, trastuzumab (TZB) and pertuzumab (PZB), as well as an antibody drug conjugate of TZB (T-DM1) are currently approved. TZB binds to domain 4 (D4) of the HER2 ectodomain (ECD)^4^, while PZB recognizes D2 close to the dimerization arm^5^, and both antibodies show synergistic effects in vitro and in vivo, as well as in the clinic^6^.

Nonetheless, the current TZB/PZB treatment only employs a single mechanism of action (MOA), that of blocking the interaction between HER2 and HER3 (in liganded and unliganded form), and is only transiently effective to block HER2 function, which does not lead to apoptosis^7^. This is one reason for resistance to arise, which was previously shown to develop following prolonged antibody treatment^8^ due to a variety of mechanisms, and it remains a major clinical challenge today.

While numerous other targets have come into focus of the biotech and pharmaceutical industry^9^, the targeting of HER2 has not yet reached its full potential. Because of its nature as a druggable disease driver and the marked overexpression on the target tissue, HER2 remains an excellent candidate to develop and study antibodies that are more advanced and have multiple MOAs. Bispecific antibodies allow additional functionality in one single molecule in comparison to conventional mAbs^10^.

Recently, the structural requirements for inducing apoptosis in HER2-addicted tumors were elucidated, using the DARPin scaffold^11^ that can also be engineered in many different orientations and formats^12,13^. Biparatopic DARPin–DARPin fusions were identified, which confine two HER2 molecules in an inactive conformation by intermolecular bridging of domains 1 and 4 (ref. ^14^) and such trapping of the receptors results in a pan-HER inhibition and triggers apoptosis^7^. The pan-HER2 inhibition leads to an OFF state of the HER2 oncogenic signaling network, similar to the one obtained with kinase inhibitor ARRY380 in combination with TZB^15^. Finally, extensive single-molecule studies revealed that trapping these receptors in large geometrically defined complexes which are strongly immobilized allows one to achieve the extraordinary cytotoxic activity of such engineered binding molecules^16^. Inspired by these results, we developed the concept to transfer the apoptosis-inducing MOA of such non-antibody molecules to an IgG scaffold.

In this work, we provide an account of the design process of bispecific and biparatopic antibodies, their thorough mechanistic study and systematic optimization for efficacy. Our final molecule, a scFv-extended IgG fusion of a particular composition and geometry, causes additional MOA components beyond receptor trapping: HER2 is being actively internalized and degraded and hence tumor cells are deprived of their most potent oncogenic signaling powerhouse.

## Results

### Engineering biparatopic scFv–Fab fusions guided by a DARPin blueprint

First, we asked if the DARPin-induced inactive, apoptosis-inducing conformation of HER2 is also achievable with an antibody-derived scaffold. Following our molecular and structural understanding of the highly active DARPin–DARPin biparatopics^7,14^, formats close to the DARPin blueprint were explored. Several DARPin-scFv and scFv-scFv tandem fusions were tested with scFv fragments derived from the antibodies A21 (ref. ^17^) and TZB as DARPin substitutes. Initially, we permuted the binders, their orientations, the linker in between and the VH/VL arrangement. The resulting molecules reduced the viability of HER2-dependent cancer cell lines as measured by diminished metabolic activity in XTT assays. A number of these constructs were comparably potent to the most active DARPin–DARPin fusion 6L1G (Supplementary Fig. 1a). Importantly, cells were also driven into apoptosis, as indicated by PARP cleavage (Supplementary Fig. 1b).

**Fig. 1:**
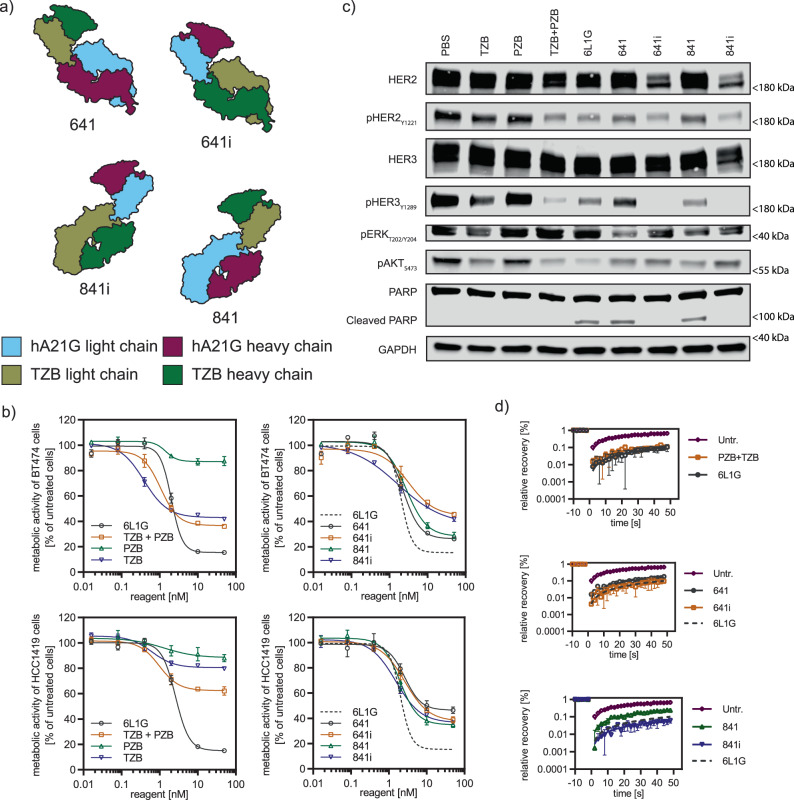
Antibody-derived biparatopic binders achieve high antiproliferative activity, induce apoptosis and partial lockdown of HER2. **a** Structural design of four closely related scFv–Fab fusions (641, 641i, 841, and 841i). They follow the blueprint of the biparatopic 6L1G, by targeting domains 1 and 4 of HER2 with either the scFv or Fab fragment. These are derived from TZB (domain 4 binder) and A21 (domain-1 binder), with the color codes indicated. **b** Proliferation inhibition (XTT assay) of HER2-addicted cancer cell lines BT474 (top row) and HCC1419 (bottom row (*n* = 3 biological replicates, mean plus error bar SD) upon treatment with the scFv–Fab variants from (**a**) was similarly strong but not equal to the DARPin–DARPin fusion 6L1G. The more active variants 641 and 841 surpassed the clinically used mAb combination TZB + PZB. All data are from the same experiment, but distributed over two plots to avoid overcrowding; 6L1G is shown again as a dotted line as reference. **c** 641 and 841 treatment (50 nM) of BT474 cells led to marked decrease of phosphorylation of key players in the HER2-dependent signaling pathways and induction of apoptosis, as seen by PARP cleavage already after 24 h in immunoblots. **d** Mobility reduction of surface HER2 analyzed with FRAP on cells (*n* = 5 cells, error bar SEM) was strong, while an incomplete “lockdown” was seen upon treatment with apoptotic agents 641 and 841 compared to DARPin 6L1G (dotted line as reference) and mAb co-treatment (TZB + PZB) (all agents 50 nM); untr. Untreated. Source data are available in the Source Data file.

Subsequently, based on this preliminary success, we increased the complexity of our fusion constructs. Four closely related scFv–Fab fusions, termed 641, 641i, 841, and 841i, were created (Fig. 1a and Supplementary Fig. 1c). For this set, the murine antibody A21 part was humanized (Supplementary Figs. 1d and 2a), and we shortened the linker, connecting the two binders, to two amino acids (glycine and serine), since shorter linkers had shown superior activity (Supplementary Fig. 2b). While exploring different formats, we observed, however, side products being produced from the expression culture, which appeared to be individual light chains and dimers thereof. Henceforth, we reengineered the Fab interface to minimize the occurrence of these side products. To this end, mutations previously used to facilitate correct light/heavy chain pairing in asymmetric bispecific IgGs^18^ were used here to abolish formation and thus secretion of these side products (Supplementary Fig. 2c, d).

We next determined the crystal structure of unliganded 841 to a resolution of 2.2 Å (Supplementary Table 1 and Supplementary Fig. 2e). This allowed us to verify that the charged residues of the designed mutations are indeed engaging in interactions and are in proximity of around 3 Å, as designed (Supplementary Fig. 2f). Note that engineered scFv–Fabs, such as 841, must bind intermolecularly: deduced from the known crystal structures of the constituent antibody-HER2 complexes of TZB and A21 (refs. ^4,17^), the binder termini are ~140 Å apart, if placed on a single HER2 molecule, yet are connected by a two amino-acid GS linker (Supplementary Fig. 2g). Since a two amino acid linker of GS can only span at most about 4 Å and the HER2 ECD had been proven rigid^19^, intramolecular binding can be excluded.

The designed scFv–Fabs strongly reduced proliferation of both TZB-sensitive BT474 and TZB-insensitive HCC1419 cells (Fig. 1b). Constructs 641 and 841 furthermore exceeded the antiproliferative activity of the combination of TZB and PZB, but they did not reach yet the antiproliferative effect of 6L1G, the biparatopic anti-HER2 DARPin–DARPin fusion. To verify that the MOA was similar, we dissected the HER2-dependent oncogenic signaling in BT474 cells 24 h post treatment by immunoblotting (Fig. 1c). We observed a marked decrease of phosphorylation of HER2 and HER3 as well as of the PI3K/AKT and ERK signaling pathways, and we furthermore detected PARP cleavage as a sign of apoptosis after 6L1G, 641, and 841 treatment.

We have previously shown that clustering of HER2 into inactive complexes, which we term “lockdown,” is required for the mechanism leading to the apoptotic response upon DARPin treatment^16^. Thus, we next investigated also the lateral mobility of HER2 on the cell surface of BT474 cells using fluorescence recovery after photobleaching (FRAP). Interestingly, 641 and 841 treatment decreased mobility within the cell membrane drastically; however, these scFv–Fab fusions did not induce a complete immobilization despite their high antiproliferative effect and the induction of apoptosis (Fig. 1d).

### Tetravalent IgG achieves complete HER2 lockdown with antiproliferative activity beyond the combination of its parental antibodies

**Fig. 2:**
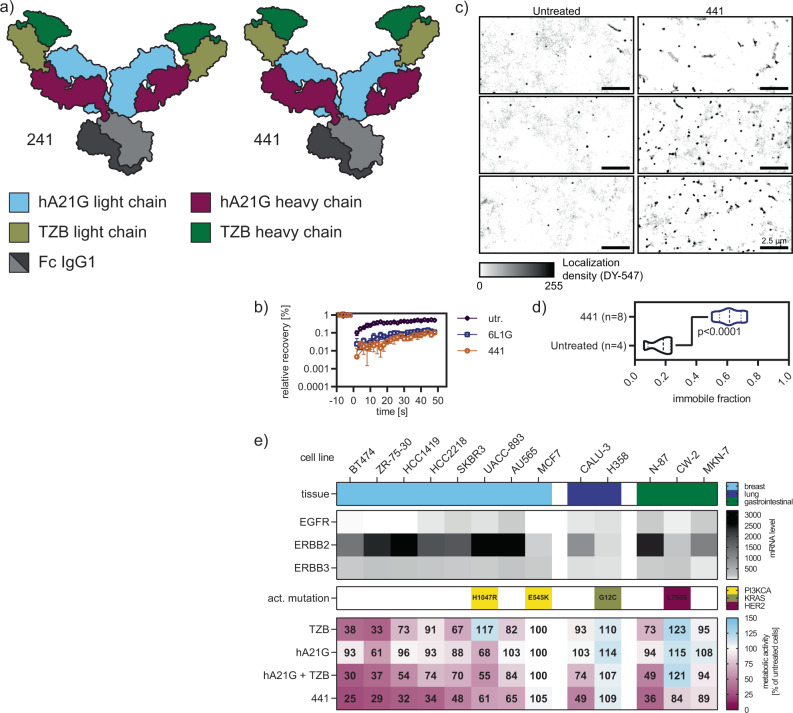
Enhanced lockdown capacity and antiproliferative activity of scFv–IgG constructs. **a** Layout of tetravalent biparatopic constructs in the scFv–IgG format, based on scFv–Fab fusions 841 and 641. Construct 241 lacked effect compared to 441 (see Supplementary Fig. 3b) **b** FRAP of 441 (50 mM) treated cells (*n* = 5 cells, error bar SEM) showed complete HER2 lockdown; untr untreated. **c** The effect of 441 on HER2 was further studied with single-molecule tracking experiments. Labeled Hela cells overexpressing a SNAP-tag HER2 fusion were analyzed with a TIRF-SMLM setup at room temperature. **d** The immobile fraction of HER2 was calculated to be 60% when analyzing single-molecule localizations of HER2 with the adapted DBSCAN algorithm. Two-sided unpaired *t* test with Welch’s correction demonstrated a significant difference from untreated cells (PBS only). **e** The additional antiproliferative activity of 441 over its parental antibody combination is seen across different cell lines and tissues (mean of *n* = 3), with relevant mutations indicated. The inhibitory effect of TZB, hA21G, and their combination on proliferation was only strong on certain HER2-dependent cell lines. 441, however, showed stronger reduction of viability throughout and thus a more universal response, hence indicating a benefit over the combination treatment. Source data are available in the Source Data file.

The scFv–Fab fusions showed a DARPin-like MOA. They reduced the mobility of HER2 strongly. Yet, the activity of the DARPin–DARPin fusion 6L1G was not achieved. To further increase the activity, we constructed a full IgG with fused scFvs, yielding the tetravalent molecules 241 and 441 (which are extensions of the scFv–Fab fusions 641 and 841, respectively) (Fig. 2a and Supplementary Fig. 1d). We hypothesized that these molecules benefit from avidity and thus improve the activity even further. Yet only one of the two, 441, the scFv fusion to the light chain, showed higher antiproliferative activity (Supplementary Fig. 3a, b), illustrating the critical importance of exact geometry for the activity.

Next, we analyzed the effect on mobility of HER2 on the surface of BT474 cells by FRAP as before. Almost no recovery at all was observed over 30 s, and hence HER2 lockdown induced by 441 was complete (Fig. 2b). We further reconstructed super-resolution images from single-molecule localization data and dramatic immobilization was evident (Fig. 2c). Using an adaptation of the DBSCAN clustering algorithm^20^, the immobile fraction of HER2 was calculated to be around 60% (Fig. 2d) and in line with our recent data for the DARPin–DARPin fusion^16^.

While monovalent DARPins targeting HER2 had practically no antiproliferative activity^14^, the bivalent mAbs TZB and A21 and especially their combination do^21^. Consequently, we compared the tetravalent construct 441 to the single treatments of TZB and hA21G IgG (h for humanized A21 mAb and G for the above specified mutations in the Fab interface) as well as their combination in a comprehensive assortment of cell lines derived from various tissues, with diverse RTK expression profiles^22^ (depicted as mRNA scores), as well as different status of clinically significant activating mutations (data taken from COSMIC^23^ and literature^24^) (Fig. 2e).

TZB and hA21G revealed only modest antiproliferative effects. In contrast, 441 was very effective on these HER2-positive cancer cell lines. As expected from a MOA on HER2 signaling, MCF7 cells (HER2 low-expressing and carrying an activating PI3K mutation) as well as H358 cells (HER2-low and carrying a constitutively active *KRAS* mutation) showed no inhibition of proliferation after treatment. In contrast, the bispecific antibody 441 showed tremendous additional benefit in all other tested cell lines independent of tissue origin, compared to the combination treatment, with the sole exception of UACC-893 cells, which carry an activating PI3K mutation. Moreover, exclusively 441 demonstrated some activity against the cell line CW-2 (low in HER2, but carrying the HER2-activating mutation L755S). The encoded HER2 mutation is most common in invasive lobular breast carcinoma with pleomorphic features^25^ and also renders tumor cells less responsive to HER2 inhibitors^26^.

### Targeting two exact epitopes in distinct arrangements results in potent constructs

Next, we wished to examine, if the higher valency alone had caused the enhanced activity. We thus investigated if biparatopic binding of 441 was still an obligatory component for its activity. For this purpose, tetravalent variants that are mono-paratopic for HER2 binding (where all four binding sites recognize the same epitope) were consequently tested next. Reduced activities were observed for variants only recognizing the A21 (termed 4A21^2^) or TZB (termed 4TZB^2^) epitope, and the latter even augmented proliferation of BT474 cells (Supplementary Fig. 3a, c). Intrigued by these findings, we reversed the orientation of the binders in 441, resulting in i441 (Supplementary Fig. 3a), for which we saw reduced activity yet again (Supplementary Fig. 3c). Together, these results emphasize again the importance of the biparatopic binding in an exact geometric arrangement.

**Fig. 3:**
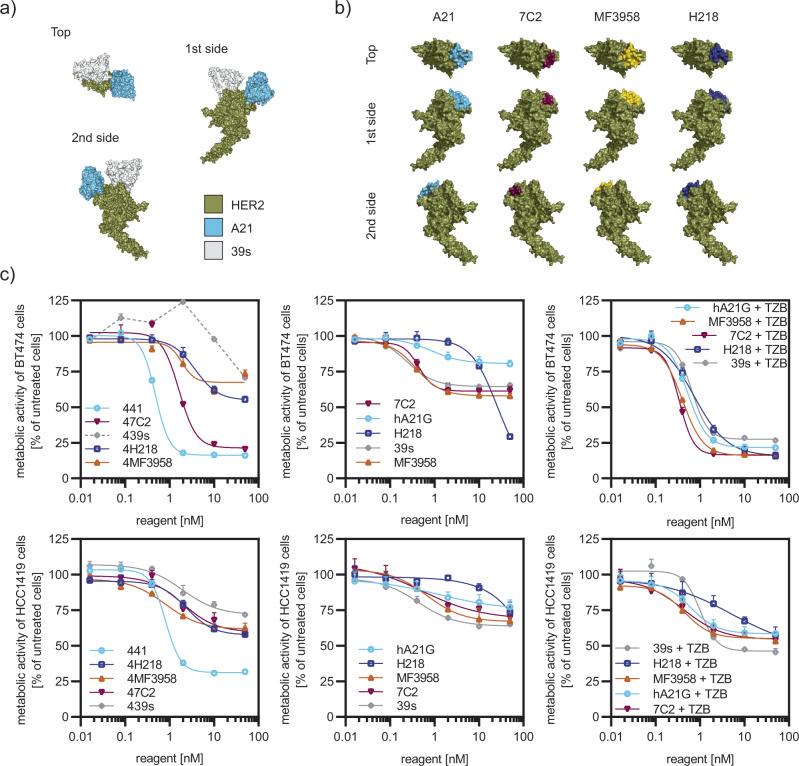
Epitope on domain I of HER2 determines activity of fusion construct. **a** Binders recognizing different epitopes on HER2 were tested as fusion partners in our format. Antibody 39s and A21 (shown as Fv fragments) recognize epitopes on top of HER2, but A21 is binding to D1 while 39s is binding to D2. **b** Crystal structures of three further antibodies complexed with HER2 (7C2, MF3958, and H2-18) were previously shown to partly overlap with the A21 epitope, but they bury less surface area upon binding (epitopes are colored). Structures were derived from PDB files 3H3B, 6ATT, 5O4G, and 3L3W and the 7C2 epitope^30^. **c** Replacement of A21 in our scFv–IgG fusion protein by any binder in (**b**) led to a reduction in antiproliferative activity of the resulting fusions (*n* = 3 biological replicates, mean plus error bar SD), tested in BT474 and HCC1419 cells. The fusions with the TZB scFv are named by “4” followed by the A21 replacement name (left panels). Note that, in the case of 39s, the fusion even augmented the proliferation of BT474 cells. Single mAbs (middle panels) were only modestly active (*n* = 3, error bar SD). Equimolar combination treatments of the mAbs with TZB (right panels) were all indistinguishable (*n* = 3, error bar SD) from each other. 441 proved superior, especially in the TZB-insensitive cell line HCC1419 (lower row). Source data are available in the Source Data file.

In the past, several anti-HER2 DARPin domain-1 binders were fused to domain 4 binders and had shown strong antiproliferative activity^7,14^. In addition, a combinatorial screening of IgGs targeting HER2 had revealed a benefit for combining binders to domain 1/2 with binders to domain 4 (ref. ^27^). We therefore tested various antibodies with known epitopes close to HER2 domain 1 as fusion partners. First, we investigated the antibody 39s, which binds to domain 2, but also in close proximity to the first domain^28^ (Fig. 3a, b). 39s is part of MEDI4276, a recently discontinued clinical stage scFv–IgG fusion drug conjugate for HER2 amplified cancers^29^. Unexpectedly, incorporation of 39s in our format led to near complete loss of activity and, surprisingly, even agonistic behavior at low nanomolar concentrations (Fig. 3c). The remaining activity at high concentrations might be attributable to the presence of two scFv TZB binders in the construct.

Next, we examined three antibodies (7C2, MF3958, H2-18) as alternatives to A21 in a biparatopic construct, as they bind to adjacent epitopes on HER2 domain 1, partly overlapping with the one of A21 (Fig. 3b). Antibody 7C2, initially claimed to bind to the very N-terminal region of HER2^30^, in fact recognizes a binding site on the A21 epitope and was shown to induce apoptosis as single agent^31^. MF3958 is the HER2-binding arm of a bispecific HER2:HER3 IgG^32^. It was shown that in this bispecific arrangement, binders to HER2 D1 were more potent in inhibiting HER3-driven growth compared to antibodies recognizing other domains of HER2. The antibody H2-18, also known as H218, which was shown to induce necrosis in HER2 tumors via activation of the RIP1–ROS–JNK–c-Jun pathway^33^, also overlaps with the A21 epitope^34^.

While the epitopes between those domain-1-binding variants are shared, A21 covers ~1820 Å^2^ of solvent-accessible surface area, compared to ~1160 Å^2^ for 7C2, 708 Å^2^ for H2-18, and 680 Å^2^ for MF3958. Exchanging A21 to these antibodies in our format led to a pronounced loss of activity for replacement by H218 (termed 4H218), MF3958 (termed 4MF3958) fusions, or by 7C2 (termed 47C2), compared to 441 (Fig. 3c). Note that, while the single IgG responses were also all weak, a combination of these IgGs with TZB as a separate IgG proved beneficial in all cases, including 39s. Yet, the 441 single-agent treatment remained superior to all combination treatments (Fig. 3c).

Finally, we investigated, if a higher affinity of the 441 binding moieties would additionally increase the efficacy or the potency. Our 441N design incorporates improved CDRs of an A21 variant with a *K*_*d*_ improved 150 times over the wild type^35^. For TZB, we took the enhanced version from a Roche patent^36^, here termed 441 R, and of course we also combined both derivates to produce 441NR. Nonetheless, neither potency nor efficacy were noticeably improved compared to 441 (Supplementary Fig. 3d).

### Biparatopic antibody interlinks and forms mega-complexes with HER2

**Fig. 4:**
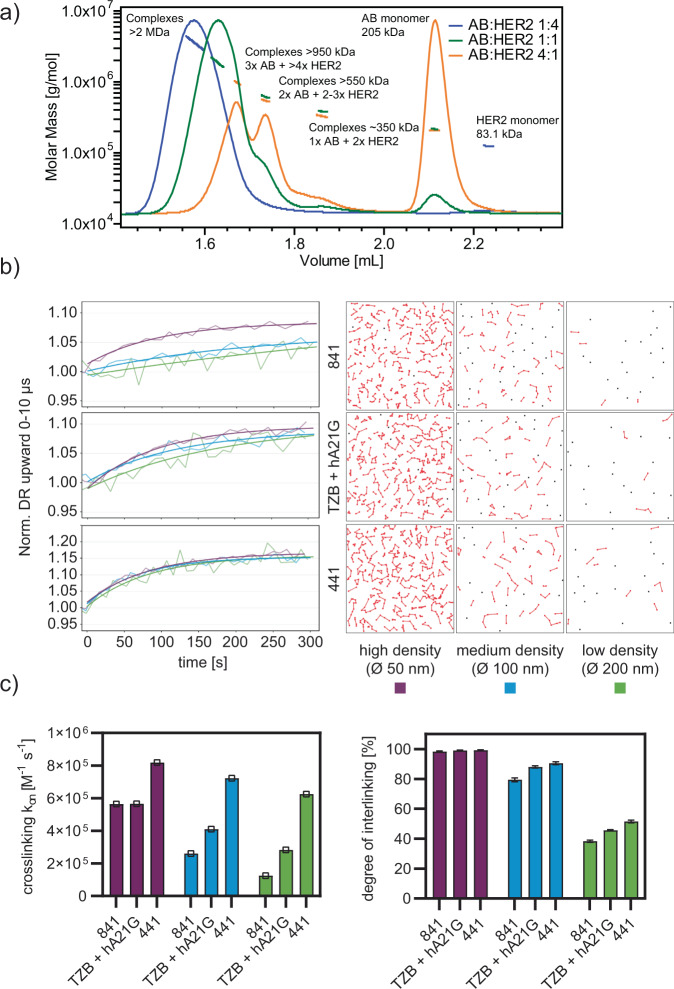
441 forms complexes with HER2 and interlinks them, facilitates internalization and ultimately degradation of HER2 clusters. **a** Multi-angle light scattering revealed that 441 formed complexes with HER2 ECD, resulting in higher-order oligomers of up to 2 MDa in size. **b** Binding studies of tetravalent 441, bivalent 841, and the antibody mixture TZB and hA21G to HER2 (see Supplementary Fig. 4 for detailed kinetic analysis) on DNA nanolevers of different densities (see colors) using the switchSENSE technology. A dependency of the on-rate on ligand density is seen for bivalent constructs (left column panels) but not for the tetravalent construct. Note that the response curves are normalized, reflecting the different number of antibodies bound at each surface density. The right-hand panels show a simulated surface, depicting a loss in interlinking of available HER2-DNA conjugates. **c** The derived association rate constants (*n* = 1, each derived from time course) are shown as well as the degree of crosslinking as calculated from the simulations in (**b**) (mean plus error bar SD, *n* = 100, from ten simulations on ten surfaces). The reduction of binding correlated well with a simulated surface, depicting a loss in interlinking of available HER2-DNA conjugates. Source data are available in the Source Data file.

Next, we aimed to gain a deeper understanding of the 441 MOAs. Therefore, we formed complexes of 441 with recombinant HER2 ECD in different molar ratios and characterized the emerging species with multi-angle light scattering. Above a molar ratio of 1:1 (441 to HER2), single populations of multi-disperse mega-complexes with up to 2 MDa in mass were recorded (Fig. 4a).

We then surveyed the complexing of 441 and HER2 in greater detail. We utilized the switchSENSE^®^ technology, which uses dynamic high-frequency electrical switching of DNA-protein conjugates and fluorescence proximity sensing for the analysis of protein interactions^37^. Analyte binding and interactions are tracked in real time through time-resolved single-photon counting of fluorophore-labeled DNA nanolevers. This technology allowed us to vary the target density, and we could therefore investigate the degree of interlinking between single HER2 ECD nanolevers by analyzing deviations in switching speed. Simultaneously, fluorescence proximity sensing was performed to measure actual binding kinetics of the analyte to HER2 without being affected by crosslinking effects (Supplementary Fig. 4a). We studied the tetravalent scFv–IgG 441, the bivalent scFv–Fab 841, as well as the combination of TZB and hA21G (Fig. 4b left panel).

Changes in switching speed reflect the degree of crosslinking. Heavily crosslinked surfaces displayed paradoxically an increased switching speed, which can be explained by the reduced switching amplitude, hence, leading to a fast “trembling effect” of the nanolevers. At reduced target density, binding and especially crosslinking events became less frequent and therefore switching speeds were again slowed down. When lowering the target density, 841 showed the most pronounced decrease in crosslinking rates, while the mAb combination and 441 were still able to maintain interlinking at lowest ligand densities. We thereafter simulated the percentage of nanolever crosslinking events, defined as at least two nanolevers being interlinked by the analyte, at the three different mean distances (Fig. 4b right panel). The simulations stochastically distributed the DNA with a length of 32 nm in a defined area. Subsequently, the length of the HER2 ECD (ca. 8 nm) was added and the span distances between the binders of the molecules 441 (ca. 20 nm), 841 (ca. 2 nm), and TZB and hA21G IgGs (ca. 14 nm) were probed as crosslinker (Fig. 4b right panel). These simulations supported the measured data and the degree of crosslinking very well (Fig. 4c). Higher mean distance reduced predominately the interlinking of 841, while even at the lowest density still around 50% of DNA molecules were bridged by 441 in simulations. Overall, the data suggest that extensive interlinking of even fairly sparsely distributed HER2 molecules is possible with 441.

### Cell surface HER2 is internalized and degraded by biparatopic antibodies

**Fig. 5:**
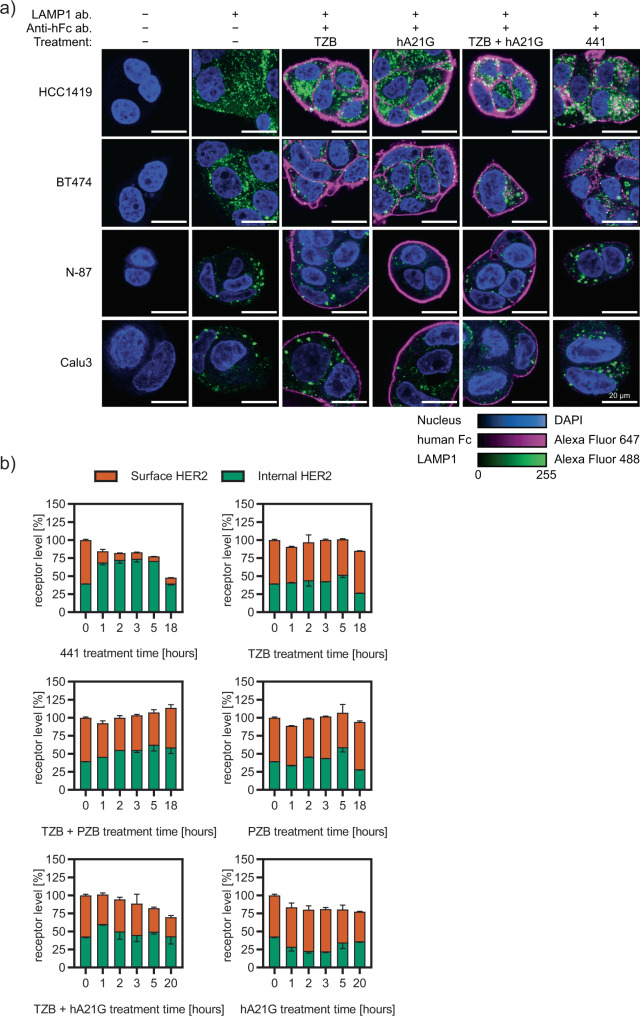
441 induces internalization and degradation of HER2 clusters. **a** Qualitative confocal microscopy studies in four different cell lines of different compounds 4 h posttreatment. Continuous membrane staining and retained membrane localization is seen for all mAbs and their combination, expect 441, which is clustered on the surface and internally in lysosomes. Human Fc regions are colored magenta, the lysosomal marker LAMP1 in green and DAPI as nuclear stain in blue. **b** Internalization and degradation were quantitatively resolved in a time-dependent manner using the recently developed SPIDA protocol^39^ (mean plus error bar SD). 441 led to distinct internalization as early as after 1 h, and thereafter internal HER2 was continuously degraded. In contrast, the combination treatment of mAbs leads to a slow gradual decrease of surface HER2, consistent with recycling inhibition without prior intracellular accumulation. Source data are available in the Source Data file.

Since we had observed the interlinking and complex formation by 441, and since the sizes of complexes of RTKs, crosslinked by combinations of mAbs, had previously been suggested to correlate with internalization^38^, we studied the effect on internalization of cellular HER2. First, the localization of 441 and its components as individual IgGs were studied 4 h posttreatment on HER2-positive cancer cells (HCC1419, BT474, N-87, and Calu-3). The single mAbs (TZB and hA21G) were localized at the surface with an even distribution, while the co-treatment led to some clusters and some continuous membrane stain (Fig. 5a). In contrast, huge complexes were formed for all cell lines upon treatment with 441, and they uniformly seemed to be internal. In ascertaining the compartment, internal 441 was found to co-stain with LAMP1, which served as a lysosomal marker (Fig. 5a).

Thereafter, we directly followed the fate of the target HER2 with another assay. Our homogenous Surface Protein Internalization and Degradation Assay^39^ (SPIDA) allows to monitor internalization and degradation of HER2 simultaneously and quantitatively. The single mAbs as well as the combination of TZB plus PZB left HER2 localization unaltered (Fig. 5b) over 18 h, in agreement with the literature^39^. Our tetravalent construct 441, on the other hand, led to pronounced induced internalization followed by a second step, active degradation. The individual antibody hA21G had essentially no effect, while the TZB + hA21G combination slightly and gradually decreased surface HER2. This is consistent with recycling inhibition of these combinations (Fig. 5b). Recycling inhibition was previously also described for a mAb combination with nonoverlapping epitopes directed against EGFR^40^.

Note that 441 shows no pronounced hydrophobicity (Supplementary Table 2) or thermal instability (Supplementary Table 3) and, therefore, the observed very high internalization and degradation rate was not attributable to an increased aggregation propensity, but is due to the particular arrangement of its binding sites on domains 1 and 4 of HER2. In summary, we discovered that 441 showed additional MOAs, subsequent to the surface clustering, not seen before in the anti-HER2 DARPin fusions^7,14^, namely an efficient induction of target internalization plus degradation.

### Biparatopic antibody 441 induces apoptosis in various cancer cells

**Fig. 6:**
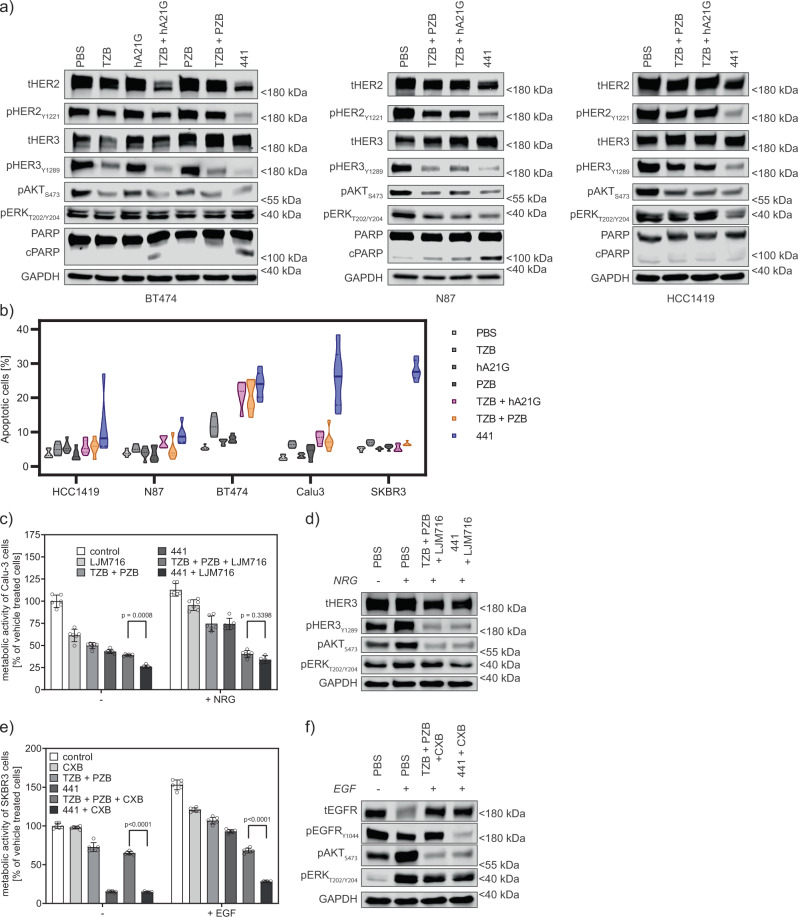
441 induces apoptosis and ablates ligand-driven growth in combination with co-receptor-targeting mAbs. **a** Twenty-four hours posttreatment, single mAbs did not reduce phosphorylation of HER2, but only of downstream signaling proteins in BT474, N-87, and HCC1419 cells as revealed by immunoblotting. Only 441 strongly reduced phosphorylation of HER2. Both 441 and TZB + hA21G additionally led to cleavage of PARP in the first two cell lines. In contrast, the TZB + PZB combination was not able to induce marked apoptosis. **b** Induction of cell death measured by dead-cell staining (PI-positive) as a percentage of total cell staining by membrane-permeable Hoechst-33342. Forty-eight hours posttreatment cells were stained (PI/Hoechst) and analyzed on a Lionheart HT-microscope. Treatment with 441 showed a significant increase of the dead-cell population over any other treatment across five different HER2-addicted cancer cell lines (*n* = 6, two-sided ANOVA with Dunnett’s correction for multiple comparison). **c** Ligand-stimulated cells pose a particular challenge for antibodies. LJM716 was shown to inhibit HER3-ligand-driven growth^41^. NRG-stimulated Calu-3 cells were kept in check at least equally well by 441 + LJM716, compared to the triple treatment of TZB, PZB, and LJM716 (*n* = 6 biological replicates, mean plus error bar 95% confidence interval, two-sided ANOVA with Tukey’s correction for multiple testing). **d** Combinations inhibit phosphorylation of HER3 and downstream kinases as shown in immunoblots. **e** Cetuximab (CXB) recognizes part of the EGF binding site on EGFR^44^ and the 441-CBX combination was significantly more beneficial in the presence of the ligand than the cocktail of TZB, PZB, and CXB (*n* = 6 biological replicates, mean plus error bar 95% confidence interval, two-sided ANOVA with Tukey’s correction for multiple testing). **f** Immunoblots show only the 441-CXB combination prevents the phosphorylation of EGFR and hence ablates downstream signaling via AKT and ERK. Source data are available in the Source Data file.

Having now characterized the multimodal MOA, we addressed the cell fate under treatment in more detail. For induction of apoptosis in HER2-addicted cancer cells, dephosphorylation of both HER2 and HER3 are required^7^. Immunoblots of BT474 cells treated for 24 h revealed a marked decrease of both phosphorylated HER2 and HER3 for 441 as well as for the combination of TZB and hA21G (Fig. 6a). Moreover, the total HER2 quantity was lowered after 441 treatment, as expected from the SPIDA data (see above). In contrast, TZB in combination with PZB as well as the single mAbs left the total level of HER2 unaltered. We detected cleaved PARP for 441 and the combination treatment, a sign of induced apoptosis. Similar responses were present in N-87 and HCC1419 cells. Most importantly, we detected cleaved PARP strongly above background apoptosis in N-87 cells. Next, we analyzed the number of viable cells and dead cells (differentiating apoptotic versus necrotic) in a subset of the responsive cell lines by HT microscopy. 441 treatment induced significantly more cell death across the panel of HER2-overexpressing cancer cell lines tested after 48 h compared to any of the other treatment (Fig. 6b).

### Biparatopic antibody remains active in ligand-driven cancer cells

Ligand-driven growth of EGFR family-dependent tumors is frequently governed by neuregulin (NRG) and EGF stimulation. HER3, the cognate receptor of NRG, is recurrently upregulated in response to HER2-directed treatment^7^. Therefore, anti-HER3 antibodies have been pursued as single agents or in combination therapy^41–43^ to counteract this effect. The antibody LJM716 locks HER3 in an inactive conformation. Thus, it keeps the receptor available for NRG binding, but incapable of dimerization with HER2^41^. 441 combined with the HER3 “locker” indeed reversed the augmented proliferation of NRG stimulation in Calu-3 cells to the same level as the triple combination of TZB + PZB + LJM716 (Fig. 6c). These treatments blocked the HER3 hyperphosphorylation seen after NRG addition and thus also the activation of the downstream signaling players (Fig. 6d).

The mAb cetuximab (CXB) partially recognizes the EGF-binding epitope on EGFR. It was further shown to sterically prevent the extended conformation compulsory for EGFR dimerization^44^. We used SKBR3 cells, which show elevated expression of EGFR (see Fig. 2e) and stimulated EGFR phosphorylation by addition of 1 nM EGF. We observed that 441 is as effective as the combination of TZB plus PZB in stimulated cells. However, 441 benefited more from the addition of CXB, compared to the TZB/PZB combination (Fig. 6e). Again, ligand-induced phosphorylation and signaling were ablated by the combination treatments (Fig. 6f). In summary, the strong action of 441 can be further augmented by the addition of anti-HER3 or anti-EGFR reagents. The resulting effect is stronger than a triple combination (two antibodies against HER2 and the additional anti-EGFR or anti-HER3 agent).

### Differential gene expression analysis in N-87 cancer cells

**Fig. 7:**
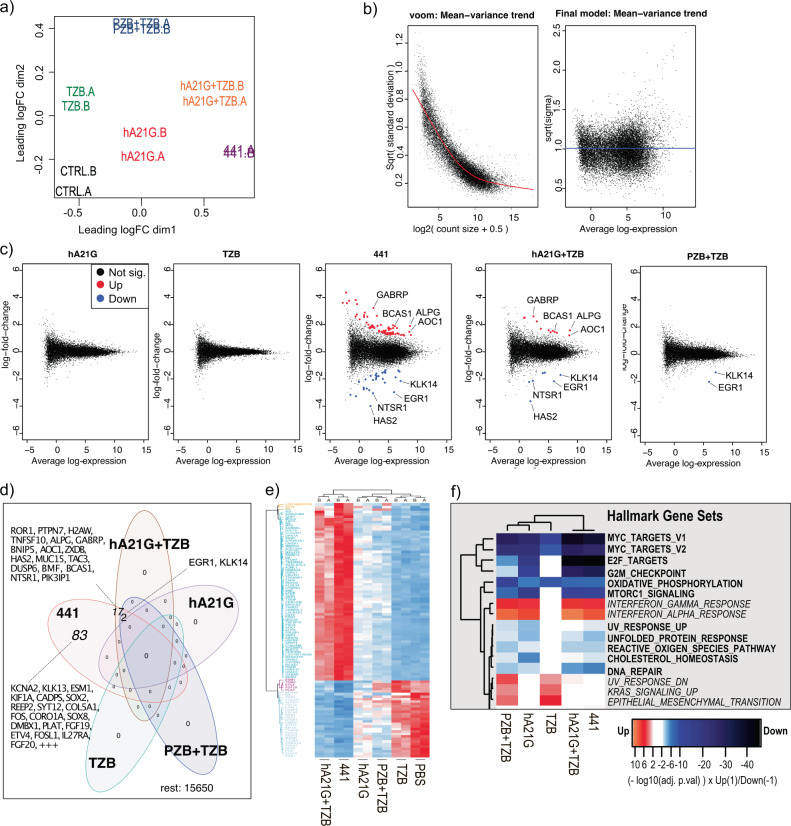
Differential gene expression analysis in N-87 cancer cells after anti-HER2 treatments by RNA-seq reveals distinct gene up- and downregulation caused by 441. **a** MDS plot for indicated treatment in biological replicates (A/B). **b** Limma/voom fit of log_2_ transformed counts per million reads (CPM) and calculation of quality weights for individual samples. **c** MDplot of significant DEG’s versus PBS control treatment. **d** Venn diagram analysis of significant DEG’s with twofold change (log_2_FC > 1). **e** Euclidean clustered heat map based on *z*-scores of significantly regulated transcripts after the 441 treatment across all indicated treatments. **f** Euclidean clustered heat map of hallmark gene sets after indicated treatments. Note, adjusted *p* values were −log_10_-transformed and downregulated gene sets were multiplied by −1 to account for negative effect. Source data are available in the Source Data file.

We further characterized the differences in antibody combination treatments (TZB + PZB and TZB + hA21G) and compared them with the biparatopic scFv-IgG 441. We performed an RNA-sequencing (RNA-seq) experiment to identify differentially expressed genes (DEGs) in N-87 cells after 48 h of treatment. As seen in the exploratory multidimensional scaling (MDS) plot (Fig. 7a), biological replicates (A/B) displayed high similarities throughout all treatments and clustered together. The MDS plot shows robust separation of the 441 treatment from the PBS control treatment on the first dimension (dim1), which attests that 441 is the main driver of variance in this experiment, hence, yielding most strongly and significantly regulated DEGs after the biparatopic scFv-IgG treatment. As can be assumed from the binding to identical epitopes, the hA21G + TZB combination treatment displayed the highest similarity to 441 in the MDS plot. The difference in antiproliferative (Figs. 2e and 3c) and proapoptotic activity (Fig. 6a, b) is also the smallest between the two. On the other hand, TZB single-agent treatment showed almost no separation from the PBS control on dim1, which is in agreement with the relatively low effect of TZB on N-87 cell proliferation (Fig. 2e), but instead it showed separation on dim2. Furthermore, the PZB + TZB combination treatment showed strongest separation from PBS control treatment on dim2.

Next, we estimated the mean-variance trend (Fig. 7b) across all treatments using the edgeR and limma-voom packages in Bioconductor, calculated log_2_-fold changes for all treatments against the PBS control treatment and generated mean-difference plots of significant DEGs versus PBS control treatment (Fig. 7c). As expected from the MDS plot, 441 displayed quantitively most DEGs across all treatments, followed by hA21G + TZB and PZB + TZB combination treatments.

Venn diagram analysis revealed increasing overlap of DEGs starting with the PZB + TZB treatment, followed by the hA21G + TZB combination treatment, and finally the 441 single-agent treatment display unique DEGs (Fig. 7d). 441 and the two different mAb combination treatments showed significant downregulation of the early growth response protein 1 (*EGR1*) and the protease kallikrein-14. *EGR1* was previously identified as a predictor for TZB resistance in gastric cancer^45^, potentially by protection from induction of apoptosis^46^. Here, we show that downregulation of *EGR1* coincided with the sensitivity of N-87 cells to all three different treatments. TZB + hA21G overlapped for a further 17 genes with 441, while 83 genes were exclusively found after 441 treatment.

We next constructed a Euclidean clustered heat map based on *z*-scores of significantly regulated transcripts after the 441 treatment across all indicated treatments (Fig. 7e). The heat map displayed that downregulated genes after 441 treatment were tendentially similarly effected after the hA21G + TZB combination treatment, albeit to a lower degree. Interestingly, TZB single-agent treatment clustered together with the PBS control treatment. Finally, we performed a gene set enrichment of the Hallmark gene set from the Molecular Signature Database using Camera^47^ (Fig. 7f). 441 induced significant downregulation of *Myc* target genes, which is in agreement with its strong antiproliferative effect on N-87 cells. Furthermore, we found significant enrichment of *KRAS* signaling upregulation after the TZB monotherapy and the PZB + TZB combination, which is in line with previous findings^7^, showing that compensatory RAS signaling activation can effectively compensate for HER3 blockage in *ERBB2*-dependent cancers after the TZB treatment.

In conclusion, we identified a rather small set of strongly regulated DEGs after all treatments in N-87 cells. 441 displayed the quantitatively strongest effect on gene expression levels. The mAb combination of TZB + hA21G was most like 441, but acted slightly more weakly, in agreement with recognition of the same epitopes and an only somewhat reduced efficacy. In general, treatments that showed a strong effect on cell proliferation consistently displayed stronger effects on gene expression.

### Single-agent 441 internalized and degraded HER2 in vivo, leading to tumor regression

Before advancing to in vivo studies, spheroids of tumor cells grown in 3D were investigated, since they provide a more realistic model of drug sensitivity than 2D cultures^48^. To this end, cells were seeded in poly-HEMA-coated round wells to allow growth independent of plastic anchorage. BT474, HCC14191, and N-87 cells formed tumor spheroids, and all were equally or more responsive to 441 than to TZB + hA21G or to TZB + PZB (Supplementary Fig. 5a).

Next, the half-life of 441 in mice was determined, and found to be 45 h (Supplementary Fig. 5b) and thus comparable to a closely related format, the DVD-IgG, for which the half-life in mice was also reported to be about 67 h^49^.

**Fig. 8:**
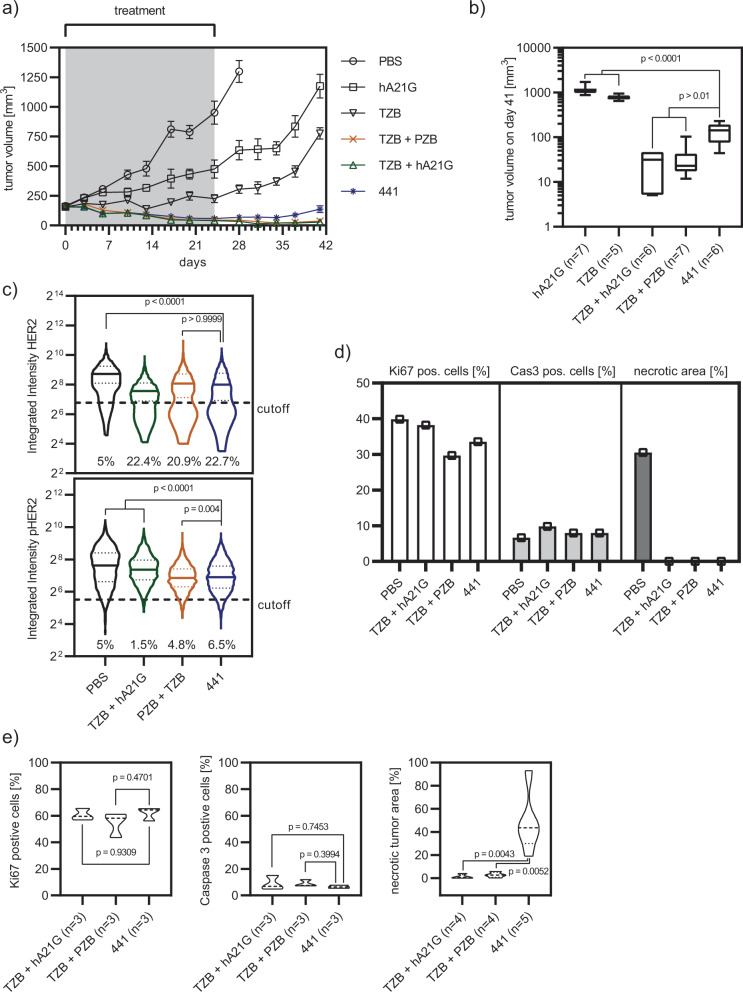
441 equally effectively controls growth of N-87 xenograft tumors in mice, compared to mAb combinations. **a** 441 and the mAb combinations showed powerful reduction of tumor volume under treatment (441 *n* = 6, TZB *n* = 5, hA21G *n* = 7, TZB + PZB *n* = 7, TZB + hA21G *n* = 6, PBS *n* = 7, error bar SEM) in female SCID beige mice with subcutaneous N-87 tumors. **b** Tumor volumes were analyzed on day 42. Single mAb treatments were significantly different to 441. 441 was non-inferior to the mAb combinations (441 *n* = 6, TZB *n* = 5, hA21G *n* = 7, TZB + PZB *n* = 7, TZB + hA21G *n* = 6, box plot with min/max whiskers, two-sided Brown-Forsythe and Welch’s ANOVA with Dunnett’s 3T multiple testing correction, adjusted *p* value 441 vs. hA21G < 0.0001; 441 vs. TZB + hA21G 0.0322; 441 vs. TZB < 0.0001, 441 vs. TZB + PZB 0.0455). **c** After the first three interventions, scouts were sacrificed and their tumors analyzed by immunohistochemistry. Effective treatments showed significantly reduced HER2 and pHER2 levels (*n* > 3000 cells, two-sided Kruskal–Wallis test with the Dunn’s multiple comparisons test). **d** Further histology revealed that loss of the tumor driver HER2 reduced the number of Ki67-positive cells (*n* > 20000 total cells), induced apoptosis (*n* > 40,000 total cells), but showed no necrosis compared to the PBS sample. **e** All tumors eventually did outgrow. Evaluation of remaining tumor mass 8 weeks post the last treatment revealed significantly more necrotic tissue for 441-treated tumors, while numbers of Ki67- and caspase-3-positive cells were equal compared to combination treatments (*n* = 3–5 mice with each >20,000 cells analyzed, two-sided ANOVA with Dunnett’s multiple comparisons test). Source data are available in the Source Data file.

We next established subcutaneous N-87 xenografts on the flanks of female SCID beige mice. This immuno-deficient model allowed us to study a HER2-dependent human xenograft. Overall, the single mAbs were ineffective in controlling the tumor growth. In contrast, 441 and mAb combination treatments led to pronounced tumor regression (Fig. 8a and Supplementary Fig. 5c). 441 and the combination treatment cohorts displayed no significant difference in tumor size (Fig. 8b and Supplementary Fig. 5d). To study if the additional MOAs of 441 established in cell culture is also found in vivo, tumors of mice treated for three injections were excised and analyzed for loss of HER2 and pHER2. All successful treatments indeed reduced the level of HER2 significantly compared to the control.

The combination of TZB and hA21G lowered the median of HER2 abundance significantly compared to 441 and TZB + PZB; however, the population of HER2-low cells was comparable across the three treatments (Fig. 8c). When studying pHER2, we noticed that TZB plus hA21G significantly reduced the levels compared to the control, but only mildly in comparison to 441 as well as TZB + PZB. 441 lowered the summed HER2 intensity by 35% and the summed pHER2 intensity by 40% compared to the PBS control. With vanishing HER2 and pHER2 levels, we also observed a decrease of Ki67-positive cells, signs of apoptosis (as caspase-3-positive cells) and no necrotic tissue compared to PBS (Fig. 8d and Supplementary Fig. 6).

Nonetheless, all initially effective treatments showed some regrowth after treatment had stopped. The remaining tumor masses 8 weeks after the last injection were studied more closely. We completed necropsy and histopathology examinations. All neoplastic nodules were densely cellular, well demarcated, nonencapsulated and expansile, and formed multiple lobules composed of neoplastic cells arranged in acini, tubules or solid clusters separated by abundant fibrous stroma. Neoplastic cells showed marked anaplastic features such as severe anisocytosis and anisokaryosis with large vesicular nuclei containing prominent nucleoli. Tumors treated with 441 showed significantly more multifocal to large focal extensive areas of necrosis, often located in the center. In contrast, the number of Ki67- and caspase-3-positive cells were indifferent from the combination treatments (Fig. 8e), consistent with similar proliferation and apoptosis rates. Of note, complete necropsy and histological examinations of the main organs were performed in at least two animals per group and the presence of metastatic lesions was ruled out.

### 441-treated tumors and cell lines derived thereof are lower in HER2 expression, but remain responsive to HER2 interference

**Fig. 9:**
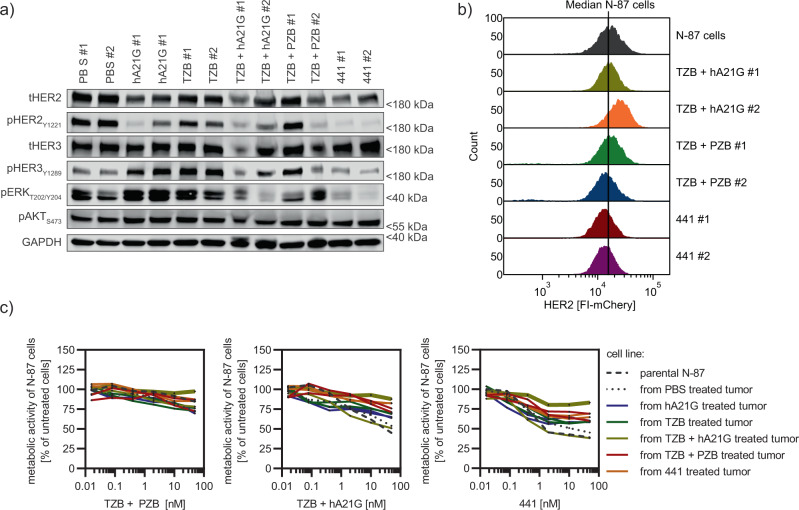
441-treated tumors and derived cell lines show reduced HER2 expression, but remain responsive to anti-HER2 treatment. **a** Lysed xenograft tumors taken from the final sacrifice, analyzed by immunoblotting, revealed severe reduction of total HER2 (tHER2), pHER2, pHER3, and pERK for 441-treated animals. mAb treatment-induced hyperphosphorylation of HER3 and ERK compared to PBS samples. mAb combinations revealed mixed responses. **b** Flow cytometric analysis of surface HER2 of cell lines established from mouse tumor material showed a loss of around 20% median intensity for 441-treated tumors. In contrast, HER2 levels stayed the same or were even augmented for TZB plus hA21G-treated animals. TZB and PZB induced a slight loss. **c** Cell lines derived from treated mouse tumors were challenged with indicated agents in a viability assay (*n* = 3 biological replicates, mean plus error bar SD, connected points, no fit). 441 was still able to reduce the proliferation the most among all studied agents. Tumors derived from 441-treated animals were also responsive to treatments; however, one TZB + hA21G-derived cell line had become practically resistant to treatments (thick dark-yellow line). Source data are available in the Source Data file.

During our study, the extracted tumor material was analyzed by immunoblotting at the point of too high a tumor burden for the animals (PBS and single mAbs) or the final point of evaluation of the study (441 and mAb combinations) (Fig. 9a). Increased phosphorylation of HER3 and, markedly, of ERK was observed after the single mAb treatments, which may have been caused by feedback loops known to promote resistance due to incomplete HER2 inhibition, as described before^50^. The mAb combination treatments led to inhomogeneous responses, where each animal showed different levels of phosphorylation of the studied signaling hubs. In contrast, both 441-treated tumors revealed marked reductions of total HER2, pHER2, pHER3, and pERK, indicative of a loss in HER2-dependent signaling.

To further investigate this point, HER2 expression on cell lines established from the tumor samples were analyzed by flow cytometry. The mAb combination had led in one case to increased HER2 expression. The three remaining cases studied remained the same or revealed slightly reduced levels of HER2. Of note, we observed a few percent of cells with little to no HER2 expression for both cell lines derived from TZB + PZB-treated tumors. These HER2 non-expressing cells were, however, morphologically different (Supplementary Fig. 7a) and stained positive for vimentin (Supplementary Fig. 7b), and hence are probably of fibroblast origin. For the two cell lines derived from 441-treated mice, the median in fluorescence was shifted by 20% to lower expression.

To analyze whether the established cell lines were still druggable by HER2 intervention, we treated them again with all agents used in the in vivo study. One cell line derived from a TZB + hA21G-treated mouse had lost responsiveness to all treatments but 441. 441 was still able to reduce proliferation by 20% (thick green line, Fig. 9c). This cell line was derived from the tumor showing loss of pHER2, but elevated HER2 expression (see above). Of note, the second cell line derived from another mouse treated with the hA21G and TZB combination had kept HER2 expression and responsiveness. 441 still demonstrated a strong response in all cell lines derived from the other treatments as well as 441, albeit a light loss in drug efficiency (Fig. 9c and Supplementary Fig. 7c). Overall, the data revealed modest reduction of HER2 expression for 441-treated tumors with slight loss of responsiveness, however suggesting that the tumors are still druggable by HER2 intervention and hence remain HER2-dependent.

## Discussion

Antibodies are nowadays a major class of drugs and on the rise particularly for cancer interventions^3^. Bispecific antibodies hold great promise for patients in the future. In this study, we designed and engineered biparatopic antibodies recognizing two different epitopes in a particular geometry on the HER2 ECD that surpass the effectiveness of the standard of care for metastatic malignancies, which is a mixture of TZB and PZB. While a TZB-PZB bispecific antibody might suggest an easy path to combine the functionality and MOA of these two clinically approved mAbs, we followed a different path, which resulted in higher efficacy. We already had previously observed that the TZB plus PZB combination is lacking potency compared to the apoptosis-inducing DARPin fusions^7^. In addition, in the DVD-IgG format, fusions of TZB and PZB had only retained the activity of the single mAbs for some fusion layouts, while others even showed pronounced agonistic behavior^51^. These findings clearly show that for activity, much more is required than a simple multiple binding of HER2.

Thus, the focus here was to interlink HER2 ECD D1 and 4 with an appropriately designed IgG derivative and to induce a HER2 “lockdown” into inactive, signaling-incompetent clusters. We aimed to transfer the MOA from the anti-HER2 DARPin blueprint^14^ to an IgG-based format, which would have the additional benefit of Fc-mediated activation of effector cells and a prolonged half-life. Our first generation of scFv–Fab constructs did not show the desired complete lockdown of HER2 and had hence less antiproliferative activity than DARPin 6L1G. Nevertheless, the scFv–Fab constructs were already superior to the clinically used mAb combination TZB and PZB and induced apoptosis, regardless of incomplete HER2 lockdown. We therefore explored a strategy to raise avidity to improve the effect, without allowing, and especially not inducing, the formation of signaling-competent HER2 complexes with itself or other members of the EGFR family. HER2 mobility was indeed further reduced by these constructs of higher valency, resulting in lower remaining viability of treated cancer cells. Eventually, the tetravalent biparatopic variant 441 showed complete HER2 lockdown and the highest efficacy and potency. Importantly, only a fraction of similar constructs showed this effect, with some others even enhancing growth (see below). Therefore, the simple linking of different binding sites is clearly not sufficient to induce apoptosis. In summary, we have hence successfully transferred the anti-HER2 DARPin MOA from DARPins to the Ig fusion construct 441. We draw this conclusion predominantly from our observations of the FRAP and single-molecule tracking experiments, where we see identical behavior for 441 and 6L1G^16^, which is then reflected in the in vitro activity.

We discovered that, besides the 6L1G-like lockdown mechanism, 441 additionally includes further MOAs, namely the induction of strong internalization and degradation of HER2. As for 6L1G, the detailed binding studies of 441 revealed high-avidity interactions and interlinking of HER2, leading to exceedingly crosslinked oligomers. Furthermore, 441 formed mega-complexes with HER2 ECD in solution and led to clusters on the surface of cells but also intracellular clusters, which subsequently localized to lysosomes. Ultimately, 441 actively induced internalization and degradation of HER2, while its components as combination only inhibited recycling. In contrast, 6L1G was shown to be neither an internalizer nor a degrader^7,39^, which underlines the fact that these MOAs can be distinguished. TZB + PZB did not alter the distribution, nor the amount of total HER2. Several early studies had suggested HER2 degradation as a MOA for TZB + PZB. However, our data and recent literature indicated that little to no HER2 degradation^39^ is induced by these antibodies. Having shown that 441 neither shows low thermal stability, nor high hydrophobicity nor any intrinsic aggregation propensity, we can deduce that this internalization- and degradation-inducing property of 441 is based on the bound epitopes, their arrangement and the structural composition of this particular tetravalent biparatopic molecule. As a consequence, the biparatopic antibody 441 induced in N-87 gastric cells a distinct pattern of gene regulation after treatment, different from the mAb combinations. A strong downregulation of Myc targets was revealed, following the two MOAs of the biparatopic IgG 441. The TZB and the TZB + PZB treatment induced upregulation of KRAS-dependent signaling, which we had previously described as one escape mechanism in HER2-positive breast cancer cell lines^7^. The response of the mAb combination TZB + hA21G, which are the parental antibodies of 441, clustered closest to 441 in terms of gene regulation.

However, the antiproliferative effect of 441 also exceeded the combination of its parts, namely the two IgGs from which it was assembled. This again underlines that the effect requires a particular geometry and not just a crosslinking of HER2. The additional 83 differently up- and downregulated genes after 441 treatment, compared to the combination treatment, further underline this fact. There is thus a necessity for its biparatopic, tetravalent design, and for this particular molecular format and these particular epitopes. Therefore, the cytotoxicity was not due to valency alone, but originated from the two fused binders and their geometric arrangement. Directing one binder to a specific epitope on domain I seemed paramount to the activity of the whole construct, as A21 could not be replaced by other variable domains binding even to epitopes very close, without losing potency. In addition, the above-average large surface area covered up by binding of A21, compared to that of the three other studied anti-domain-1 candidates sharing part of the epitope, may be another key factor explaining the better performance. Nonetheless, since the heavy chain fusion (241) as well as the inverse variant (i441) also fell behind against 441, another major determining pillar must be the geometrical arrangement of its binding components, and therefore the resulting cluster of receptors and bound antibodies.

Importantly, neither the activity of the single antibodies nor that of their combination predicted their activity in bi-epitopic targeting. All anti-domain-1 mAbs and the anti-domain-2 mAb 39s worked well in combination with TZB, yet in our fusion format only fusion with the component A21 exceeded the respective combinations. The weak performance of the 39s fusion construct is in contrast to the data presented in an earlier study^29^, which had claimed high degradation for the closely related MEDI4276. However, the initially reported degradation could not be reproduced for the naked MEDI4276 molecule^39^. Receptor degradation was also seen for pan-HER, a combination of six mAbs targeting nonoverlapping epitopes on EGFR, HER2, and HER3 (ref. ^52^). A future question will thus be to test these in a direct comparison for their degradation properties, but also considering the development challenges of six antibodies versus a defined bispecific one, which can be further engineered.

The multimodal MOAs (surface lockdown of HER2 to inactive clusters and internalization, followed by degradation) abrogated proliferative signaling, led to profound apoptosis in HER2-dependent cancer cells and finally induced pronounced tumor regression in the N-87 in vivo model. Tumor-bearing mice treated with 441 demonstrated loss of HER2 and pHER2. This reduced the number of Ki67-positive cells and finally the tumor burden. In contrast, the single mAb treatments were nearly completely ineffective in blocking tumor growth of the N-87 xenograft. Conversely, the combination treatment and 441 showed initial tumor reduction, followed by regrowth after the treatment period had ended. None of the studied treatments were thus able to completely cure mice of the N-87 xenograft, using the treatment regimen studied. While the strong benefit of 441 over the mAb combinations could not be fully translated from cell culture to this particular xenograft study, the in vivo studies should be expanded in the future to define the parameters where the in vitro differentiation is best demonstrated.

A durable response of 441 might currently be somewhat weakened by the half-life of just about 2 days, which is enough for the presented proof-of-principle study, but needs improvement for subsequent clinical application. Fortunately, the great advances in increasing half-life by engineering improved FcRn interactions, in conjunction with a powerful in vitro recycling assay^53^, have made this rather straightforward. Outgrown tumors of the 441 cohort revealed a high degree of necrosis, loss of HER2, and especially pHER2. Downregulation of HER2 was shown before as escape mechanism to HER2-targeted therapy in the clinic^54^. Yet, cell lines derived of the 441 cohort remained responsive to HER2-targeted agents.

Another often observed resistance mechanism to HER2-targeted therapy is ligand-driven growth, which can be also addressed using HER2:HER3 bispecific antibodies^32^. 441 was also able to ablate some of the ligand-driven growth. However, the most significant further improvement of the activity of 441 was the addition of an EGFR or a HER3 targeting antibody. We believe that this strategy will inhibit both signaling axes, the one through heterodimers, but also through HER2:HER2 homodimers^7^.

In summary, 441 stands as a successful multi-MOA-designed protein. Nevertheless, to elucidate the complete multitude of mechanisms and to understand which mechanism contributes to tumor regression to which proportion, additional, more detailed analyses of 441 will be needed in the future.

## Methods

### Cell lines and biological material

The following cell lines were purchased from ATCC: AU565, BT474, Calu-3, HCC1419, HCC2218, H358, MCF7, N-87, SKBR3, UACC-893, and ZR-75-30. Cell lines CW-2 and MKN-7 were obtained from RIKEN Biobank, Japan. RIKEN Biobank is the authentic source for MKN-7, a commonly misidentified cell line. Hela cells for the single-molecule tracking experiments were provided by the laboratory of Prof. J. Piehler. Cell lines were maintained at 37 °C/5% CO_2_ in a humidified atmosphere and routinely checked for mycoplasma contamination.

### Viability assays

For assays in 2D culture, cells were seeded at predetermined optimal densities in 96-flat-well plates (Corning). For assays in 3D cultures, 1000 cells per well were seeded in Poly-HEMA-coated (0.6 mg per well) 96 round-well plates (Corning). In addition, cells were pelleted at 300 *g* for 3 min to facilitate more homogenous spheroid growth. Twenty-four hours post seed, compounds for treatments were added and incubated for another 96 h. XTT assay reagent (neoFroxx) was added, and after development, absorbance was read at 463 vs. 670 nm on a TECAN Infinite M1000 Pro reader. Data shown are expressed as percentage of metabolic activity compared to untreated or vehicle-treated cells, corrected by subtraction of blank values. Titration curves were fitted with a “log(inhibitor) vs. response—variable slope (four parameters)” fit (GraphPad Prism). Data were weighted by 1/*Y*^2^ and the Hill slopes were constrained to >“−3”. Antiproliferative effects were compared by calculating the area under the curve with a 95% confidence interval. Nonoverlapping areas were considered different. Single concentration points were compared by the two-sided Student’s *t* test or two-sided ANOVA where applicable and corrected for multiple testing where due; see individual figure captions for detailed description of applied statistical tests.

### Analysis of total and dead cells by HT microscopy

BT474, HCC1419, SKBR3, N-87, and Calu-3 cells were seeded at a density of 10,000 cells 16 h prior to treatment into-thin layer black/clear-well 96-well plates (NUNC). Cells were treated with 100 nM of indicated protein or PBS and incubated for 48 h in a standard tissue culture incubator. Afterwards, cells were stained by addition of propidium iodide and Hoechst-33342 (Life Technology) and incubated for 30 min at 37 °C prior to microscopy. Plates were analyzed on a Lionheart HT-microscope (Biotek) and quantified by the proprietary Gen5 software (Biotek). The numbers of dead cells were compared across cell lines and treatments with a two-way ANOVA with Dunnett’s correction for multiple comparison. Significance was established at *p* < 0.01.

### Western Blot

Semi-quantitative Western Blot analysis to dissect progression of HER2-dependent signaling upon interference with studied compounds was conducted as previously published^7^. In brief, cell or tumor lysates were equalized for protein content and separated by reducing 4–20% SDS PAGE. Subsequently, they were blotted onto PVDF-FL membranes (Millipore), which were incubated overnight with the indicated primary antibodies at 4 °C (for dilutions see Supplementary Table 4). Signals were generated by addition of secondary fluorescently labeled antibodies and intensities were read on an Odyssey imaging system (LI-COR Biosciences).

### Multi-angle light scattering coupled to size exclusion high-performance liquid chromatography

Antibodies and HER2 ECD (EMP Genetech) were complexed in PBS at 4 °C for 1 h at indicated molar ratios. Ten microliter thereof were injected and eluted from a BEH500 size exclusion column (Waters) attached to a 1260 Infinity II LC System (Agilent) with a flow rate of 0.3 ml/min and ultimately investigated with a µDAWN detector (Wyatt). Data analysis was carried out with the ASTRA software 6.1 (Wyatt).

### Fluorescence recovery after photobleaching (FRAP)

HER2 mobility on the surface of treated cells was analyzed by FRAP as described elsewhere^16^. Briefly, labeled affibody zHER2 was allowed to bind to HER2 on cells before the compound to be studied was added. We additionally included metabolic inhibition (50 mM sodium azide and 10 mM 2-deoxy-D-glucose in Dulbecco’s phosphate-buffered saline) to investigate HER2 immobilization uncoupled from internalization. The region of interest (ROI) was bleached and we recorded fluorescence recovery over time. Intensities in the ROI were corrected for photobleaching and relative recoveries were calculated.

### Single-molecule localization microscopy

Total internal reflection microscopy with single-molecule localization and tracking (TIRF-SMLM) was performed essentially as described elsewhere^16^. In brief, HeLa cells were transiently transfected with an N-terminal SNAP-HER2 fusion and afterwards imaged on a customized Olympus IX71 inverted microscope at room temperature, to retard internalization as long as possible. Single molecules were identified by pixel-wise hypothesis-testing against the local background and, with sub-pixel precision, localized to reconstruct super-resolution images. Immobile particles were then identified in the obtained data by a modified DBSCAN method^20^. The fractions of immobile particles (of total) were compared with an unpaired Student’s *t* test with Welch’s correction.

### Immunofluorescence

40–100,000 cells were seeded in poly-lysine-coated µ-slide 8-well chambers (ibidi). Cells were treated with agents (50 nM) for 4 h. Thereafter, wells were washed with PBS and cells fixed with 4% paraformaldehyde (PFA). Antibodies and 4′,6-diamidino-2-phenylindole (DAPI) in PBS, supplemented with 0.5% Tween-20, were incubated for 30 min at RT. After another round of washing, secondary antibodies were added also for 30 min at RT. Finally, cells were washed once more and fixed in 4% PFA. Cells were imaged on a SP5 or SP8 FALCON FLIM microscope (both Leica) and data were processed using Fiji^55^.

### Measuring apparent binding kinetics and HER2 interlinking

HER2 (Sino Biological) was conjugated to the complementary DNA single strand cNL-B96 using the amine coupling kit 7 (Dynamic Biosensors). Crosslinking and kinetics studies were conducted on a switchSENSE DRX2 instrument (Dynamic Biosensors) using a multipurpose density chip MP3 (Dynamic Biosensors) that carried three different red fluorophore-labeled DNA-nanolever densities with an average distance between the nanolevers of 50, 100, or 200 nm, respectively. The chip was functionalized with HER2 by HER2-DNA-conjugate hybridization to the nanolevers in stopped flow. The dynamic mode for hydrodynamic friction analysis was described elsewhere^37^. The association (5 min) as well as the dissociation (60 min) of the analytes 441, 841, or TZB + hA21G combination (20 nM each) were conducted at a constant flow rate of 100 µL/min and in static mode (constant −0.1-V potential) on the medium-HER2-density channel (100-nm distance) by recording the quenching effect upon analyte binding in close proximity to the red fluorophore and by referencing to a blank run (buffer only). For crosslinking analyses, an alternating potential (−0.4 to 0.4 V) with a 5-kHz frequency was applied to acquire the dynamic response upon 20 nM 441, 841, or TZB + hA21G analyte binding in a 100-μL/min flow for 5 min. The surface was regenerated with fresh HER2 conjugate before dynamic response measurements on each of the three different HER2 densities. Binding and crosslinking kinetics curves were exponentially fitted (mono- or biphasically) using the switchANALYSIS software (Dynamic Biosensors) to acquire *k*_on_ (both binding and crosslinking), *k*_off_ and *K*_*D*_.

### Simulation of HER2 nanolever crosslinking

The surface interlinking simulation consisted of three consecutive steps. At first, a square surface with a given side length (1 µm in all images) was divided into equally sized hexagons with an in-radius of 2.5 nm. Each of these areas will (potentially) hold one DNA lever at its center. The in-radius of 2.5 nm guaranteed a minimum distance between DNA levers of 5 nm, which is an approximate physical density limit based on DNA radius and electrostatic repulsion between DNA molecules. Second, each of the defined hexagons was checked for whether it should contain a DNA molecule. The probability of a hexagon containing a DNA molecule is equal to the ratio of the hexagon area and the area of another hexagon with in-radius equal to half of a given distance between DNA levers. This way, the DNA levers were stochastically distributed over the surface but maintain a given average distance. Third, the interlinking molecules were introduced one-by-one to the surface. Each molecule was bound randomly to a DNA lever with a free binding site. After the molecule’s initial binding, it bound to the closest neighboring DNA lever (with free binding site), which it could reach (including the length of both DNA levers and the molecule). If no second binding partner was found, the molecule was left singly bound. Each molecule that binds reduces the number of free binding sites of a DNA lever by one. Initially, each DNA lever starts with two free binding sites. Once all binding sites were occupied, the total number of DNA levers interlinked through bound molecules was compared to the total number of initial DNA levers. The binding of molecules was repeated ten times on ten individual surfaces. The size for each simulated surface density was chosen so that boundary effects are negligible.

### Surface Protein Internalization and Degradation Assay (SPIDA)

Distribution of surface and internal fractions of HER2 as well as the total receptor level were simultaneously quantified in flow cytometry-based experiments according to our recent SPIDA protocol^39^. In brief, HER2 was expressed as a HaloTag fusion in a HEK293 cell line. Treated cells were sequentially labeled with two fluorescent HaloTag ligands (one non-cell-permeable, the other permeable) to allow the binary distinction of localization at a chosen time point. Signals were read with a flow cytometer (Supplementary Fig. 8 shows the gating strategy) and furthermore channel rescaling was used to calculate total levels and thus detect internalization and potential degradation.

### RNA-seq sample preparation, sequencing, and mapping

N-87 cancer cells were seeded 16 h prior to treatment in complete media at a cell density of 10,000 cells per cm^2^. Cells were treated for 48 h in biological duplicates with 100 nM of indicated agents (for combination treatments, each agent at 100 nM) or PBS. Afterwards, medium was removed, cells were washed three times with ice-cold PBS (Sigma Aldrich), cells were scraped in ice-cold PBS buffer, cells were pelleted by centrifugation at 300 *g* for 3 min and, subsequently, cell pellets were snap-frozen in liquid nitrogen. Frozen pellets were sent to Genewiz (www.genewiz.com) for RNA extraction and RNA-seq. RNA extracts had to pass quality control, and mRNA enrichment was performed by polyA enrichment. Sequencing was done on an Illumina HiSeq system using a 2×150 PE HO sequencing mode with an average of 20 million reads per sample. A mean Illumina quality score above 35 was achieved and the GC distributions were close to average. Reads were trimmed by Trimmomatic v. 0.36, mapped to GRCh38/hg38 using the STAR aligner v. 2.5.2b, and raw counts were normalized to TPM values by Genewiz.

### Differential gene expression analysis

Differential gene expression analysis was performed on raw count values for 57,500 transcripts obtained from Genewiz (see Supplementary Information) in R-studio^56^ using Bioconductor^57^ and the packages edgeR^58^ and Limma-Voom^59^. The heat map was generated in Perseus^60^.

### Animal experiments

All mice experiments were performed in accordance with the Swiss animal protection law and with approval of the Cantonal Veterinary Office (Zurich, Switzerland).

### N-87 tumor study

Fifty-four to sixty-seven days old female Fox Chase SCID Beige mice (CB17.Cg-*Prkdc*^*scid*^*Lyst*^*bg-J*^/Crl; Charles River) were inoculated on the right flank with five million N-87 cells in 50% matrigel (Corning #356237; Lot 7184514). Tumor dimensions were measured with an electronic caliper and the tumor volume was approximated by *V* = 0.5 × length × width × width. When the tumors had reached a mean of ~150 mm^3^, mice were randomized. Six treatment cohorts were formed: PBS, hA21G, TZB, hA21G plus TZB, TZB plus PZB and 441. Allocated mice were treated twice per week with 10 mg/kg of antibody via tail vein injections for a total of eight interventions with a three- to four-day interval. For combination treatments, each antibody was given as a 10-mg/kg dose, so mice received 20-mg/kg antibody total. None of the studied agents did show any toxicity or induced weight loss in our animals. After completion of treatment, tumor outgrowth was followed for an additional 8 weeks. Mice having reached health exclusion criteria were sacrificed. All researchers involved in the in vivo study were blinded during the experiment for the assignment of the mice to cohorts, and tumor growth data were for the first time analyzed after all injections had been performed. 441 mean tumor volumes on days 41 and 76 were compared to other groups by the two-sided Brown-Forsythe and Welch’s ANOVA test and corrected for multiple comparison with Dunnett’s T3 test (GraphPad Prism). Two mice per treatment were sacrificed and their tumors used for immunoblotting and cell line generation. Separate mice were fixed in formalin and prepared for pathology (see below).

### Histology and immunohistochemistry examinations and morphometric analyses

N-87 tumors were established in SCID beige mice as described above. Tumors with a size >400 mm^3^ were treated with 441, TZB plus PZB, as well as TZB plus hA21G for three times in the same interval and dose regime as before. One day post the third injection, mice were perfused and tumors removed from the animals. Furthermore, animals dropping out of the main study were also prepared as follows. Their tumors with adjacent skin and subcutaneous tissue were fixed in 4% neutral-buffered formalin, embedded in paraffin wax, sectioned at 3–5-µm thickness, routinely stained with hematoxylin and eosin. Formalin-fixed paraffin-embedded tissues were also subjected to immunohistochemical examination for Ki67 (monoclonal anti-rabbit; clone 30-9, Roche; ready to use dilution, incubation 32 min at 37 °C; secondary antibody donkey-anti-rabbit, Jackson ImmunoResearch; Ventana Discovery DAB Map kit 760-124), caspase-3 Asp175 (monoclonal anti-rabbit, Cell Signaling; dilution 1:400, incubation 1 h at 37 °C; secondary antibody goat-anti-rabbit; Ventana Discovery OmniMap anti-Rb HRP 760-4311; Discovery ChromoMap DAB kit 760-159). A Discovery XT research instrument (Ventana Medical System, Inc, Tucson, Arizona, USA) was used for the Ki67 and caspase-3 protocols. The Cell Conditioning 1 pretreatment (CC1 Ventana 950-124) was used for the Discovery XT systems and antigen retrieval for all antibodies was performed by using EDTA buffer (pH 9) for the Dako autostainer.

Morphometry analyses for Ki67 and caspase 3 were performed with Visiopharm software (Visiopharm Integrator System, Version 5.0.4. 1382; Visiopharm, Horsholm, Denmark). Manual annotations (ROI) were introduced to quantify the total surface of tumor and surface of necrosis. For the analyses of immunohistochemistry, sections of ten ROIs of the tumor with intact epithelial tumor cells (without necrosis or stroma) were manually introduced. For Ki67 and caspase 3, a standard application of cell classification was run within the ROIs to count total number of cells (selected as total number of nuclei) and the number of positive cells.

Quantification of HER2 expression and phosphorylation status was performed on a single-cell level from at least ten different tumor tissue sections from the corresponding N-87 tumor xenograft study after the indicated treatments. DAPI/HER2 (dilution 1:250, Merck OP15) or DAPI/pHER2 (dilution 1:100, Cell Signaling Technology 2249) counterstained tissue slides were recorded on a LEICA SP8 confocal microscope. Microcopy pictures were analyzed in CellProfiler 2.2.0 using a primary mask to identify N-87 cancer cells based on shape, size, and intensity parameter thresholds and a secondary mask to detect HER2 or pHER2 staining intensity of the corresponding cell, respectively. Note that N-87 tumor tissue sections contained up to 50% of mouse fibroblasts, which were excluded from subsequent HER/pHER2 quantification based on the primary DAPI mask. N-87 cells were gated on DAPI intensity across different conditions, and finally 3000 cells for each condition were obtained for statistical comparison with a Kruskal–Wallis test with Dunn’s multiple comparisons test.

### Statistical analysis and reproducibility

In general, statistical analysis is described in the corresponding method (see above) or the individual figure caption. All experiments were repeated at least once with similar results, but the in vivo study and the crystallization of 841.

### Reporting summary

Further information on research design is available in the Nature Research Reporting Summary linked to this article.

## Supplementary information

Supplementary Information

Reporting Summary

Supplementary Data 1

Description of Additional Supplementary Files

## Data Availability

Coordinates and structure factors of 841 were deposited in the Protein Data Bank under Accession Code 6ZQK (https://www.rcsb.org/structure/6ZQK) with corresponding crystallographic data collection and refinement statistics shown in Supplementary Table 1. Supplementary data are provided with this paper. All data are also available upon request. Source data are provided with this paper.

## Source data

Source Data

## Code availability

Custom code is available upon request.
