# Supplementary material for: Engineering an anti-HER2 biparatopic antibody with a multimodal mechanism of action
Source: Nat Commun. 2021 Jun 18;12:3790. doi: 10.1038/s41467-021-23948-6 (PMC8213836; doi:10.1038/s41467-021-23948-6)
Supplement: Supplementary file 1 — Supplementary Information [file 41467_2021_23948_MOESM1_ESM.pdf]

Engineering an anti-HER2 biparatopic antibody with a multimodal mechanism of action

Florian Kast[1], Martin Schwill[1,6], Jakob C. Stüber[1,7], Svende Pfundstein[1,2], Gabriela Nagy-Davidescu[1], Josep M. Monné Rodríguez[3], Frauke Seehusen[3], Christian P. Richter[4], Annemarie Honegger[1], Karen Patricia Hartmann[1], Thomas G. Weber[5], Felix Kroener[5], Patrick Ernst[1,8], Jacob Piehler[4] and Andreas Plückthun[1,*]

1 Department of Biochemistry, University of Zurich, Zurich, Switzerland

2 Zurich Integrative Rodent Physiology (ZIRP), University of Zurich, Zurich, Switzerland

3 Laboratory for Animal Model Pathology, Institute of Veterinary Pathology, Vetsuisse Faculty, University of Zurich, Zurich, Switzerland

4 Department of Biology/Chemistry and Center for Cellular Nanoanalytics, University of Osnabrück, Osnabrück, Germany

5 Dynamic Biosensors GmbH, Planegg, Germany

6 present address: TOLREMO therapeutics AG, Muttenz, Switzerland

7 present address: Roche Innovation Center Munich, Penzberg, Germany

8 present address: Dean's Office and Coordination Office of the Academic Medicine Zurich, University of Zurich, Zurich Switzerland

** corresponding author*

Address of corresponding author
Department of Biochemistry
University of Zurich
Winterthurerstr. 190
8057 Zurich
Switzerland
Tel: +41 44 635 5570
FAX: +41 44 635 5712
Email: plueckthun@bioc.uzh.ch

Supplementary Methods

Reagents and chemicals

All reagents were obtained from Sigma/Merck unless stated otherwise. Trastuzumab and Pertuzumab were purchased from Kantonsapotheke Zürich. Cetuximab was a kind gift from Kerry A. Chester (University College London). Commercially available antibodies used in this study are listed in Supplementary Table 4.

Engineering and humanization of antibodies

The sequences of the A21 VL and VH domain[1] are most closely related to murine germline family consensus sequences muVK08 (81.6% identity, 83.9% similarity) and muVH01 (63.0% ident., 73.9% sim.) The closest individual germline sequences are muVK 8-30 (87.1% ident, 89.4% sim.) and muVH 1S34 (75.0% ident., 81.9% sim.). The domains were humanized by a loop graft to the closest human germline family consensus frameworks, huVK04 (75.2% ident., 81.3% sim.) and huVH01 (55.1% ident., 66.6% sim). In addition to the CDR residues, light chain residue L4 (packing against residue L27b) and L49 (adjacent to CDR L2) as well as heavy chain residues H28 (adjacent to CDR H2), H69, H71, H75 (outer loop) and H93 (adjacent to CDR H3) were retained from the original A21 sequence (all residue numbers according to KABAT numbering). Additionally, LC Q38K, LC V133K and HC Q39E as well as HC S188E (also KABAT numbering) were mutated (Supplementary Figure 2 c).

Expression and purification of studied constructs

DARPin-DARPin and scFv-DARPin fusions were expressed and purified as previously described[2]. All other proteins were transiently expressed in CHO-S cells (Thermo Fischer Scientific), cultivated in CHOgro (Mirus), from polycistronic vectors, which were transfected via a PEI based protocol[3]. Shaker cultures (ISF1-X, Kühner) were harvested 5-10 days post transfection by centrifugation and supernatants were additionally cleared by 0.22 µm filtration. Recombinant proteins were captured by Protein A chromatography and, where necessary, additionally purified by cation exchange and polished by preparative gel filtration. Purity was assessed by SDS page and monomeric content by analytical size exclusion chromatography, and in addition, masses were determined by electrospray ionization mass spectrometry where due to confirm the molecular identity.

Crystallization and structure determination of construct 841

The purified fusion protein construct was concentrated to 10 mg/mL using an Amicon® centrifugal concentrator (50,000 MWCO, Merck Millipore, Massachusetts, USA). For crystallization, 96-well sparse matrix screens (Hampton Research, California, USA and Molecular Dimensions (Anatrace, Ohio, USA)) were used to find suitable crystallization conditions with three different mother liquor-to-protein ratios (1:1, 2:1, 3:1 or 5:1 in 300-400 nL) and incubated at 4 °C against 75 µL reservoir solution. Crystals grew in various conditions within 24 h, and crystals were harvested and flash-frozen in liquid nitrogen after incubation for 5-10 s in mother liquor supplemented with 20% ethylene glycol.

Data collection was carried out at beamline X06SA (Paul Scherrer Institute, Villigen, Switzerland) equipped with an Eiger 16M detector (Dectris, Baden-Wättwil, Switzerland) at a wavelength of 1 Å. Crystallization conditions and data collection and refinement statistics are summarized in Supplementary Table 1. Data were processed with XDS, XSCALE and XDSCONV[4]; molecular replacement was used to solve the phases using PHASER[5] and the model was built in Coot[6]. Structure refinement was done using refMAC5[7], PHENIX refine[8] and BUSTER[9]. The final resolution of the datasets was determined by paired refinement in pdb_redo[10] based on ref. 11.

Thermal unfolding

PBS-buffered samples were diluted to 250 µg/ml, and SYPRO Orange (Thermo Fischer Scientific) was added (1:1000 dilution). While heating at a rate of 2°K/min, the increase in fluorescence was recorded

on a Stratagene Mx3005P real-time PCR instrument (Agilent). The apparent melting temperatures were extracted from fitting replicates from three independent measurements with a 4-parameter sigmoidal fit (GraphPad Prism).

Protein hydrophobicity characterization by analytical hydrophobic interaction chromatography (HIC)

A TSK gel butyl-NPR column (3.5 cm length, Tosoh Bioscience LLC) attached to an Agilent 1260 Infinity HPLC system was used to determine relative hydrophobicity of bispecific constructs and control IgGs. Injected samples were eluted with a linear gradient from 0 to 100% B and a flow rate of 0.8 ml/min (A: 1.5 M ammonium sulfate in 25 mM potassium phosphate pH 7; B: 25 mM potassium phosphate pH 7).

Animal experiments

All mice experiments were performed in accordance with the Swiss animal protection law and with approval of the Cantonal Veterinary Office (Zurich, Switzerland).

Mouse housing

Five animals per cage were kept in Allentown XJ (19.37 x 38.13 x 13.03 cm) cages equipped with autoclaved dust-free wooden bedding (80-90 g / cage) (Lignocell Select), nesting material (2 autoclaved paper tissues per cage) and a red plastic house (ZOONLAB GmbH). Mice had access to pelleted mouse diet (Kliba No. 3436, Provimi Kliba) and sterilized chlorinated drinking water ad libitum. The temperature was kept 23 ± 3°C and the relative humidity at 50 ± 15%. 50 complete changes of filtered air per hour (HEPA H 14 filter, IVC-system from Allentown Inc.) were carried out. The light/dark circle consisted of 12 h artificial light between 07:00-19:00 with 100-200 lux between the racks one meter from the ground.

Half-life determination of 441

3 mg of antibody per kg bodyweight were injected intravenously in female NSG mice (NOD.Cg-*Prkdc*^{scid} *Il2rg*^{tm1Wjl}/SzJ, Charles River). Two mice were bled at each indicated time point and sera were collected. Antibody concentrations were determined by sandwich ELISA. The capture antibody from mouse, specific for human Fc (JacksonImmunoResearch 209-005-098), was directly coated on Nunc MaxiSorp plates (Thermo Fischer Scientific). Bound serum antibodies and serum-spiked standards were revealed by alkaline phosphatase-conjugated goat antibody directed against human kappa chain (SouthernBiotech #2060-04). Absorbance was read at 405 nm and 540 nm on a TECAN Infinite M1000 Pro instrument. A_{540} was subtracted from A_{405}. Serum antibody concentration was interpolated with a 95% confidence interval from the standard curve and half-life data were fitted with a two-phase decay model (GraphPad Prism) with weighting of data by $1/Y^2$ and fixing final concentration to zero.

HER2 surface stain of N-87 xenograft-derived cell lines

Cells were detached, washed twice with PBS, and one million cells was incubated for 1 hour at RT with a mCherry-6L1G fusion (100 nM). Afterwards cells were washed twice with PBS, stained for live cells and fixed with 4% paraformaldehyde (PFA). Cells were analyzed with a flow cytometry (Supplementary Figure 8 shows the gating strategy).

Supplementary Figures

a)

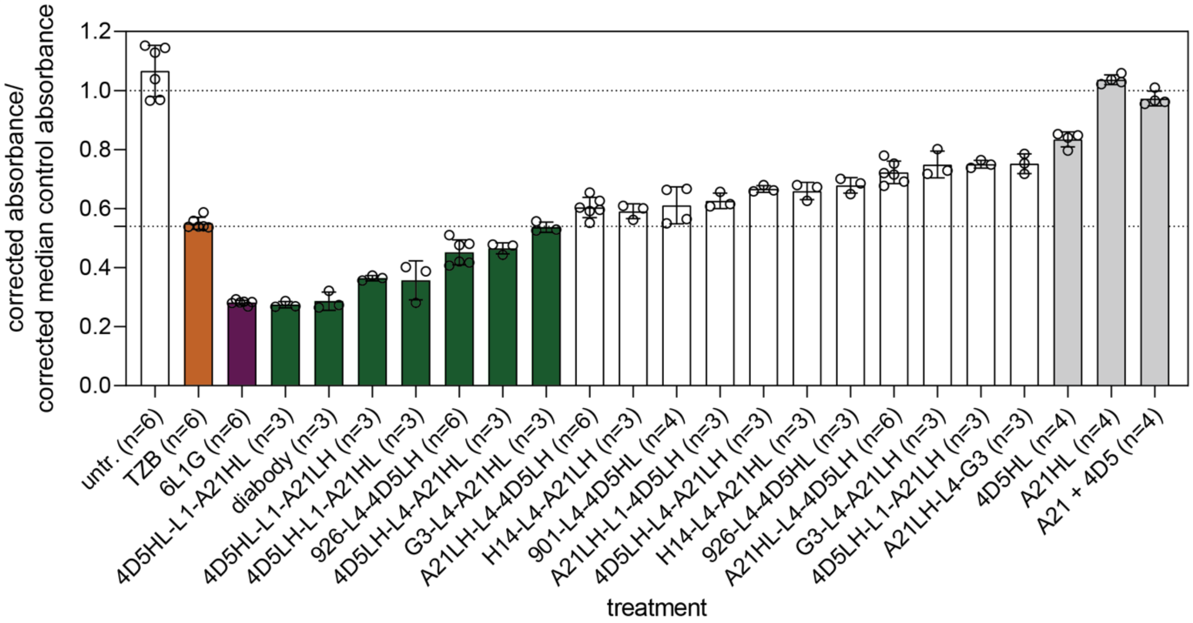

b)

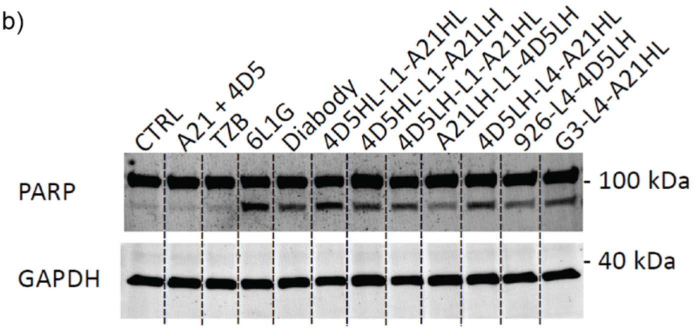

c)

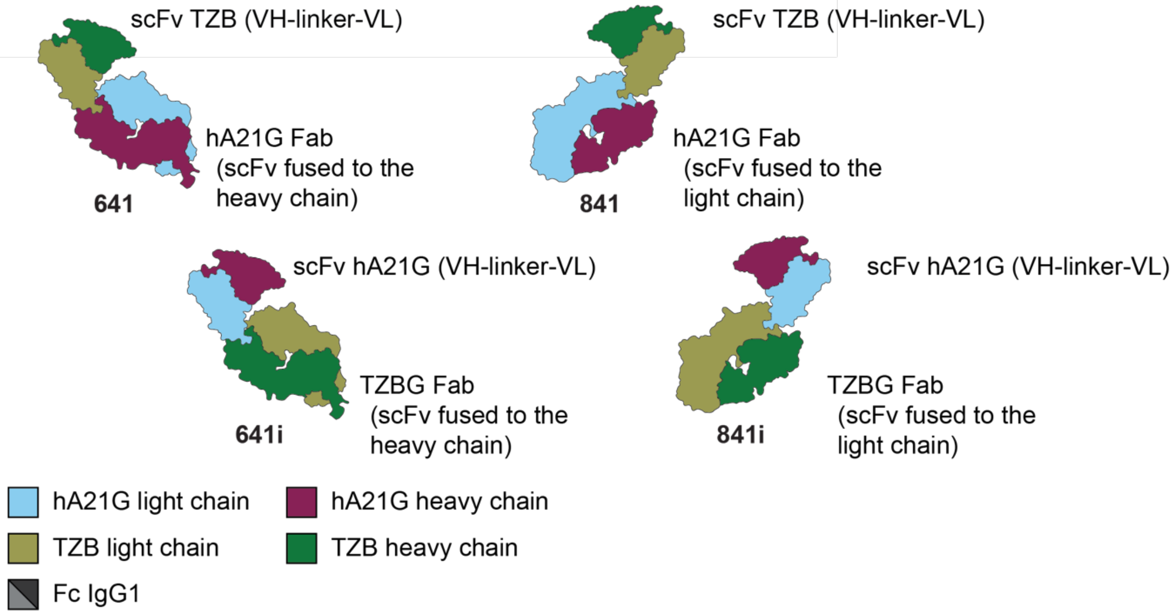

d)

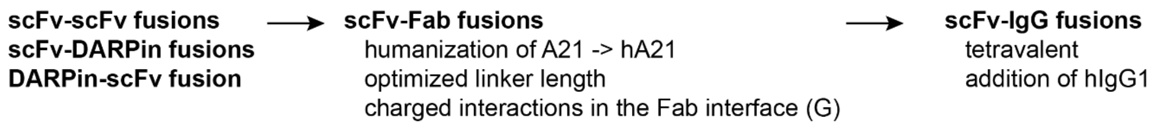

Supplementary Figure 1 – DARPin-scFv fusions and scFv-scFv fusions achieve high antiproliferative activity and induce apoptosis. Layout of scFv-Fab fusions and design process. The IgGs TZB and A21 were reformatted to scFvs (named here 4D5 and A21, black bars) and tested as agents in different fusion layouts. 6-L1-G (violet) is the apoptosis-inducing DARPin-DARPin fusion used for reference[12]. L1 denotes a GGGGS linker and L4 denotes a $(GGGGS)_4$ linker. LH and HL describe the order of the arrangement of the two variable domains in the scFv fragment. (a) Individual scFv fragments and their combinations (grey bars) had no antiproliferative effect on BT474 cells (n=biological replicates, mean plus error bar SD) in a viability assay. The TZB IgG control (not showing apoptosis) is shown in orange, the positive control 6-L1-G is shown in violet. All fusion proteins more potent that TZB are shown in green. Some genetic fusions of A21 and 4D5, but only in certain arrangements, showed almost equal maximum anti-proliferative activity compared to the biparatopic DARPin 6L1G. (b) Induction of PARP cleavage monitored by western blot after 48 hours of treatment with 100 nM of agent. The most active variants in (a), 4D5HL-L1-A21HL and 4D5LH-L1-A21HL, induced PARP cleavage to a similar extent as the biparatopic DARPin 6L1G. (c) Four closely related scFv-Fab fusions were designed. The A21 epitope is situated on domain 1 of HER2 and the TZB epitope on domain 4. (d) Three generation of constructs were tested. First generation: simple bivalent, biparatopic fusions of two binding molecules, such as DARPins and scFvs. Second generation: scFv-Fab fusions with humanized antibodies, optimized linkers and charge interactions in the Fab interface to avoid incorrect chain pairing (see below). Third generation: scFv-IgG fusions in a tetravalent layout fused to a standard hIgG1. [Source Data provided]

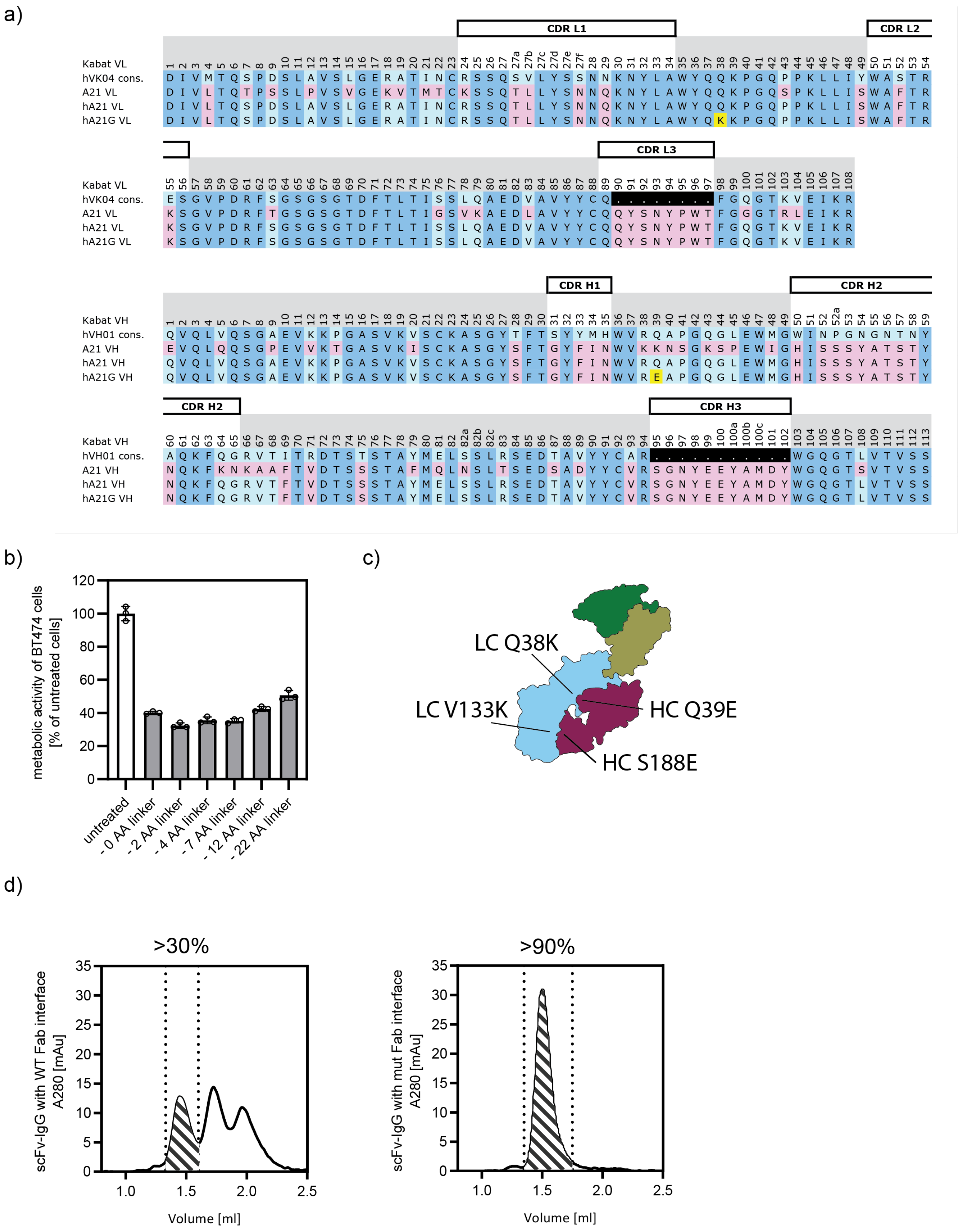
a)
CDR L1
CDR L2
CDR L3
Kabat VL
hVK04 cons. DIVMTQSPDSLAVSLGERATINCRSSQSVLYSSNNKNYLAWYQQKPGQPPKLLIYWASTR
A21 VL DIVLTQTPSSLPVSVGEKVTMTCKSSQTLLYSNNQKNYLAWYQQKPGQSPKLLISWAFTR
hA21 VL DIVLTQSPDSLAVSLGERATINCRSSQTLLYSNNQKNYLAWYQQKPGQPPKLLISWAFTR
hA21G VL DIVLTQSPDSLAVSLGERATINCRSSQTLLYSNNQKNYLAWYQKKPGQPPKLLISWAFTR
hVK04 cons. ESGVPDRFSGSGSGTDFTLTISSLQAEDVAVYYCQ........FGQGTKVEIKR
A21 VL KSGVPDRFTGSGSGTDFTLTIGSVKAEDLAVYYCQQYSNYPWTFGGGTRLEIKR
hA21 VL KSGVPDRFSGSGSGTDFTLTISSLQAEDVAVYYCQQYSNYPWTFGQGTKVEIKR
hA21G VL KSGVPDRFSGSGSGTDFTLTISSLQAEDVAVYYCQQYSNYPWTFGQGTKVEIKR
CDR H1
CDR H2
CDR H3
Kabat VH
hVH01 cons. QVQLVQSGAEVKKPGASVKVSCKASGYTFTSYYMHWVRQAPGQGLEWMGWINPGNGNTNY
A21 VH EVQLQQSGPEVVKTGASVKISCKASGYSFTGYFINWVKKNSGKSPEWIGHISSSYATSTY
hA21 VH QVQLVQSGAEVKKPGASVKVSCKASGYSFTGYFINWVRQAPGQGLEWMGHISSSYATSTY
hA21G VH QVQLVQSGAEVKKPGASVKVSCKASGYSFTGYFINWVREAPGQGLEWMGHISSSYATSTY
hVH01 cons. AQKFQGRVTITRDTSTSTAYMELSSLRSEDTAVYYCAR...........WGQGTLVTVSS
A21 VH NQKFKNKAAFTVDTSSSTAFMQLNSLTSEDSADYYCVRSGNYEEYAMDYWGQGTSVTVSS
hA21 VH NQKFQGRVTFTVDTSSSTAYMELSSLRSEDTAVYYCVRSGNYEEYAMDYWGQGTLVTVSS
hA21G VH NQKFQGRVTFTVDTSSSTAYMELSSLRSEDTAVYYCVRSGNYEEYAMDYWGQGTLVTVSS
b)
metabolic activity of BT474 cells [% of untreated cells]
0
20
40
60
80
100
120
untreated
- 0 AA linker
- 2 AA linker
- 4 AA linker
- 7 AA linker
- 12 AA linker
- 22 AA linker
c)
LC Q38K
LC V133K
HC Q39E
HC S188E
d)
>30%
scFv-IgG with WT Fab interface A280 [mAu]
Volume [ml]
>90%
scFv-IgG with mut Fab interface A280 [mAu]
Volume [ml]
0
5
10
15
20
25
30
35
1.0
1.5
2.0
2.5

e)

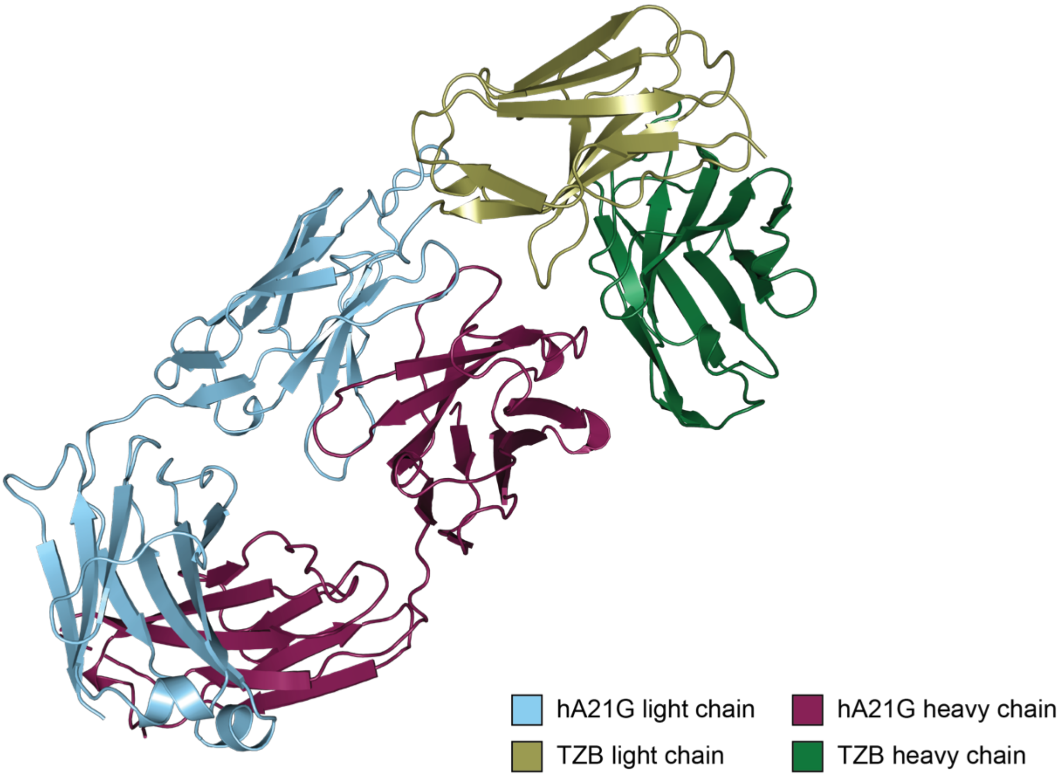

f)

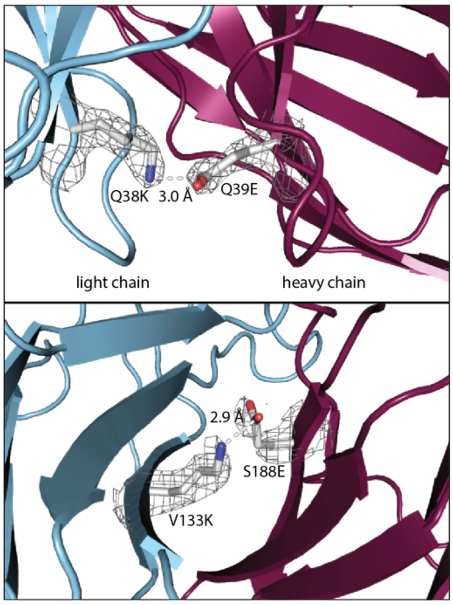

g)

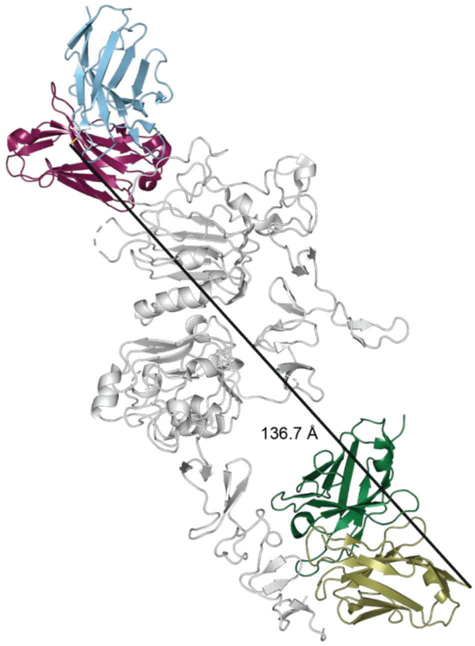

Supplementary Figure 2 – scFv-Fab fragments were engineered for multiple aspects before in-depth characterization (a) Humanization of A21. Sequence alignment of the humanized A21 sequence to the sequences of the acceptor frameworks (consensus sequences of the human Vkappa 04 and VH 01 germline families) and the A21 CDR donor sequence[1]. Sequence positions that agree between the CDR donor and the acceptor framework are colored pale blue, sequence positions that differ are colored pale cyan for the human sequence and pink for the A21 sequence. Positions highlighted in yellow are mutations introduced to prevent chain mispairing (see below). (b) XTT assays showed shorter linkers

between the scFv and Fab fragment improved antiproliferative activity with an optimum at two amino acids (GS) (n=3, mean plus error bar SD). (c) Additional mutations in the Fab interface were incorporated to reduce the amount of side products produced. (d) Variants without (left) and with (right) mutations seen in (c) were loaded on an analytical SEC column directly after Protein A purification. The yield of desired product was improved from around 30 to above 90%. (e) Crystal structure of the scFv-Fab construct 841 (f) The mutations in the Fab interface are interacting as designed. The residues that guide dimerization are around 3 Å apart (2Fo-Fc and contour level 1σ). (g) The final product 841 must bind intermolecularly: the termini of binders are more than 135 Å apart when placed on a single HER2 molecule, and the GS linker may only span about 4 Å. HER2 with liganded TZB Fab (PDB ID: 1N8Z) and liganded scFv A21 (PDB ID: 3H3B), both shown as scFvs, were combined in a single model, and the distance was measured from the C-terminus of the scFv of TZB to the N-terminus of the scFv A21. [Source Data provided]

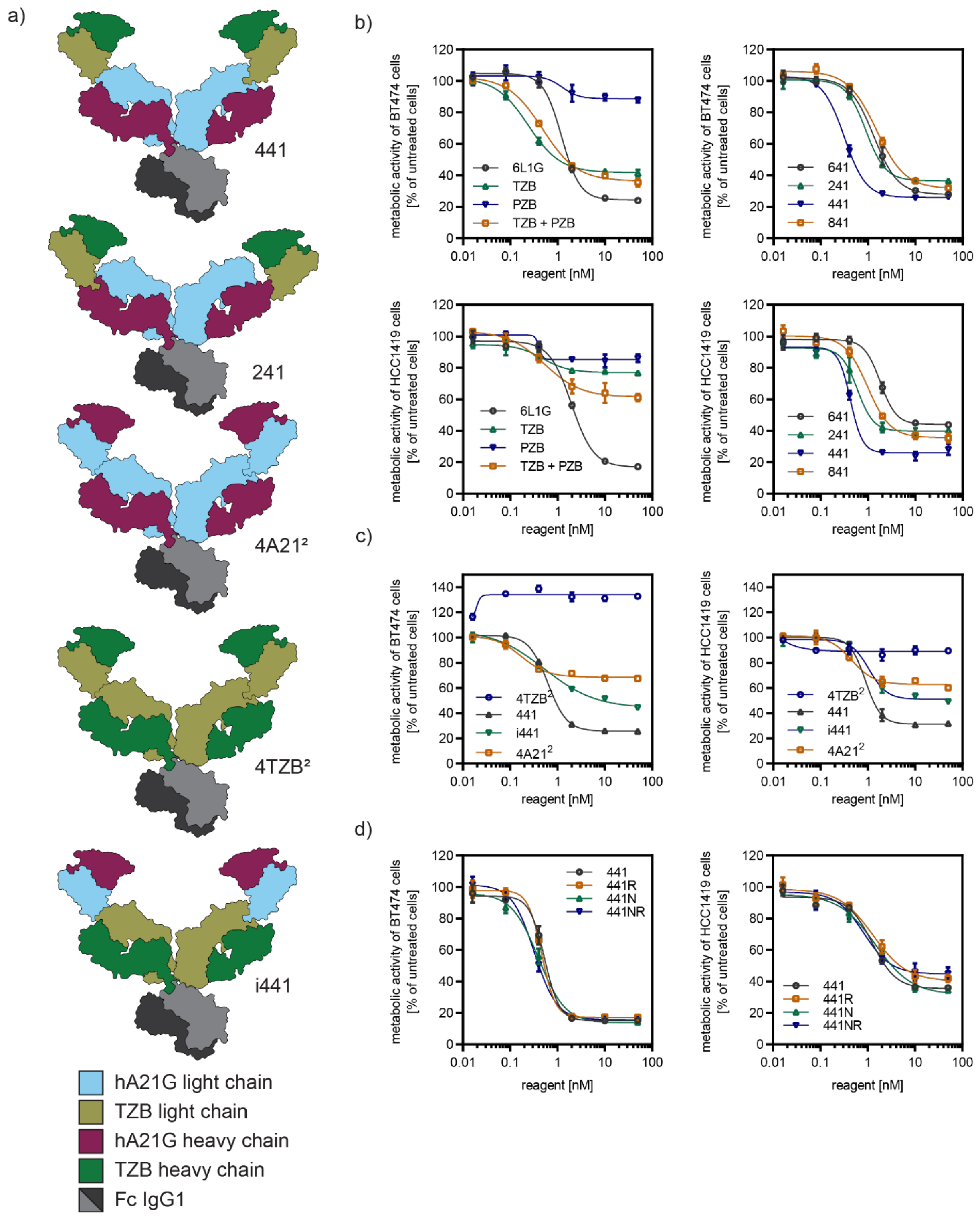

Supplementary Figure 3 –Only 441 achieves high antiproliferative activity among scFv-IgG fusions compared to control constructs. (a) Layout overview of tetravalent fusions. 241 (based on the scFv-Fab 641, layout see (a)) is the corresponding scFv fusion to the heavy chain, while 441 is a scFv fusion to the light chain. As a further control, molecules were also made, in which all binding sites were identical, denoted as the name of the parent "squared", 4A212 and TZB2. Binder orientation was further reversed in the variant i441. (b) the scFv-IgG fusion 241 did not show the same antiproliferative activity as 441, neither in BT474 nor in HCC1419 cells, even though it consists of the same binding components (n=3, mean plus error bar SD). (c) All further variants were inferior to 441 in terms of

antiproliferative effect, and 4TZB2 even showed increased proliferation (n=3, mean plus error bar SD). (d) Improved affinity variants (for details, see text) for A21 nor for TZB did not improve potency and/or efficacy compared to 441 (n=3, mean plus error bar SD). [Source Data provided]

Medium density (100nm)

841

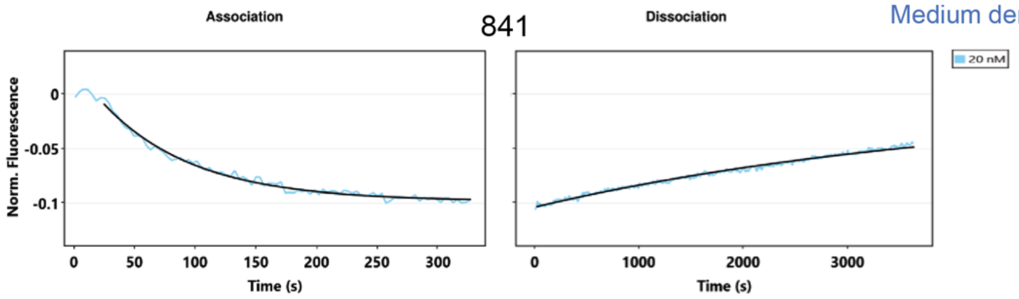

k_{on} = 6.53 ± 0.20 x10^5 $M^{-1}\cdot s^{-1}$ k_{off} = 2.40 ± 0.01 x10^{-4} s^{-1} k_D = 368 ± 11 pM

441

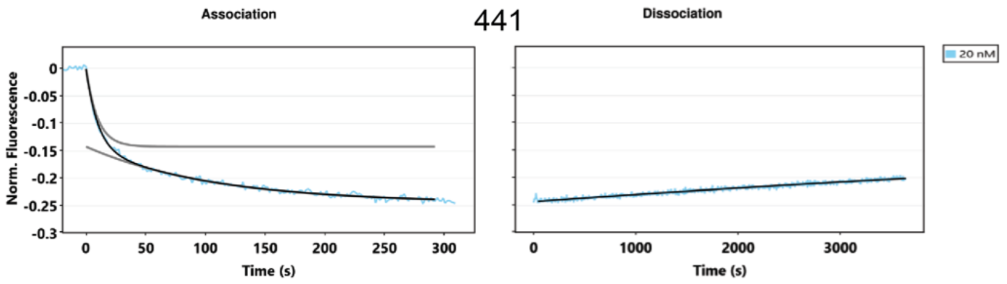

k_{on1}= 5.19 ± 0.27 x10^6 $M^{-1}\cdot s^{-1}$

k_{on2}= 4.65 ± 0.31 x10^5 $M^{-1}\cdot s^{-1}$

k_{off} = 8.05 ± 0.06 x10^{-5} s^{-1}

k_{D1} = 15.5 ± 0.8 pM

k_{D2} = 173 ± 12 pM

hA21G + TZB

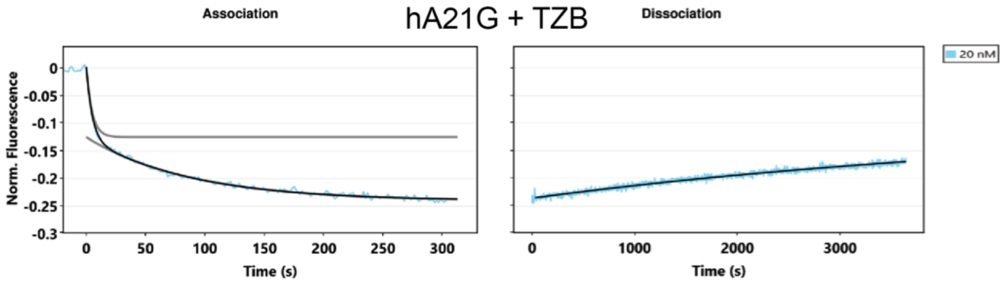

k_{on1}= 1.03 ± 0.06 x10^7 $M^{-1}\cdot s^{-1}$

k_{on2}= 5.69 ± 0.19 x10^5 $M^{-1}\cdot s^{-1}$

k_{off} = 1.83 ± 0.01 x10^{-4} s^{-1}

k_{D1} = 17.8 ± 1.0 pM

k_{D2} = 322 ± 11 pM

hA21G

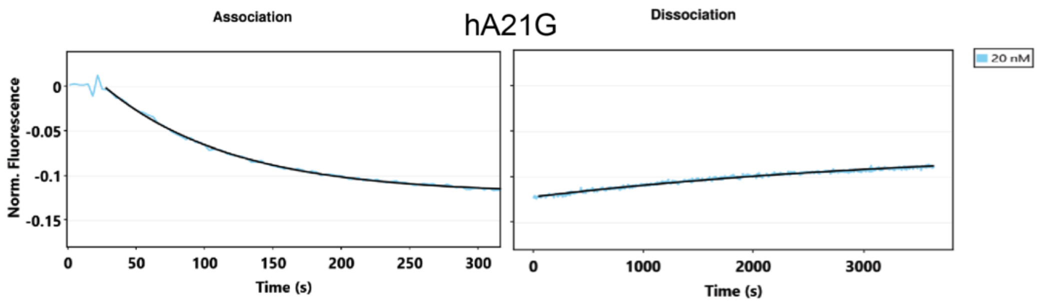

k_{on} = 5.23 ± 0.08 x10^5 $M^{-1}\cdot s^{-1}$ k_{off} = 2.68 ± 0.01 x10^{-4} s^{-1} k_D =512 ± 8 pM

TZB

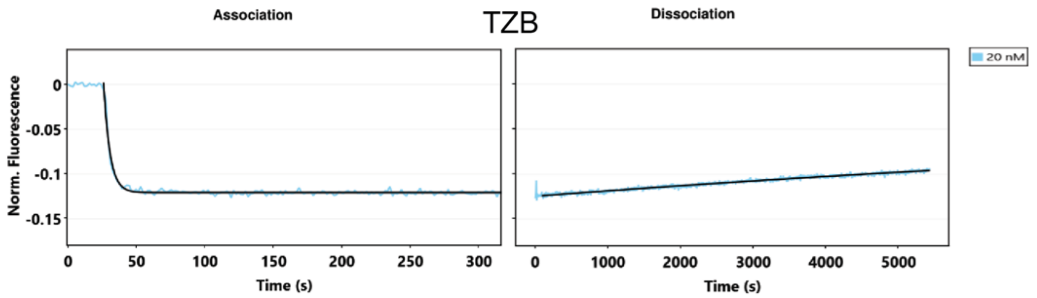

k_{on} = 1.08 ± 0.03 x10^7 $M^{-1}\cdot s^{-1}$ k_{off} = 6.01 ± 0.62 x10^{-5} s^{-1} k_D =5.57 ± 0.60 pM

Supplementary Figure 4 – 441 binds to HER2 with high avidity. The fluorescence proximity sensing measurement mode of the switchSENSE® technology was used to study binding to HER2, independent of the influence of interlinking. TZB was demonstrated to bind in the single-digit picomolar realm to HER2, while A21 was around 500 pM. The scFv-Fab fusion 841 showed a monophasic on-rate and it bound to HER2 with an avidity of around 350 pM. Binding of 441 as well as the combination of TZB and hA21G was very comparable and showed biphasic on-rate behavior (fast component close to 107 M-1 s-1 and slower one 5x105 M-1 s-1). Interestingly, the off rates were monophasic for both cases, resulting in an avidity of 20 pM for the fast component and around 200-300 pM for the slower part. [Source Data provided]

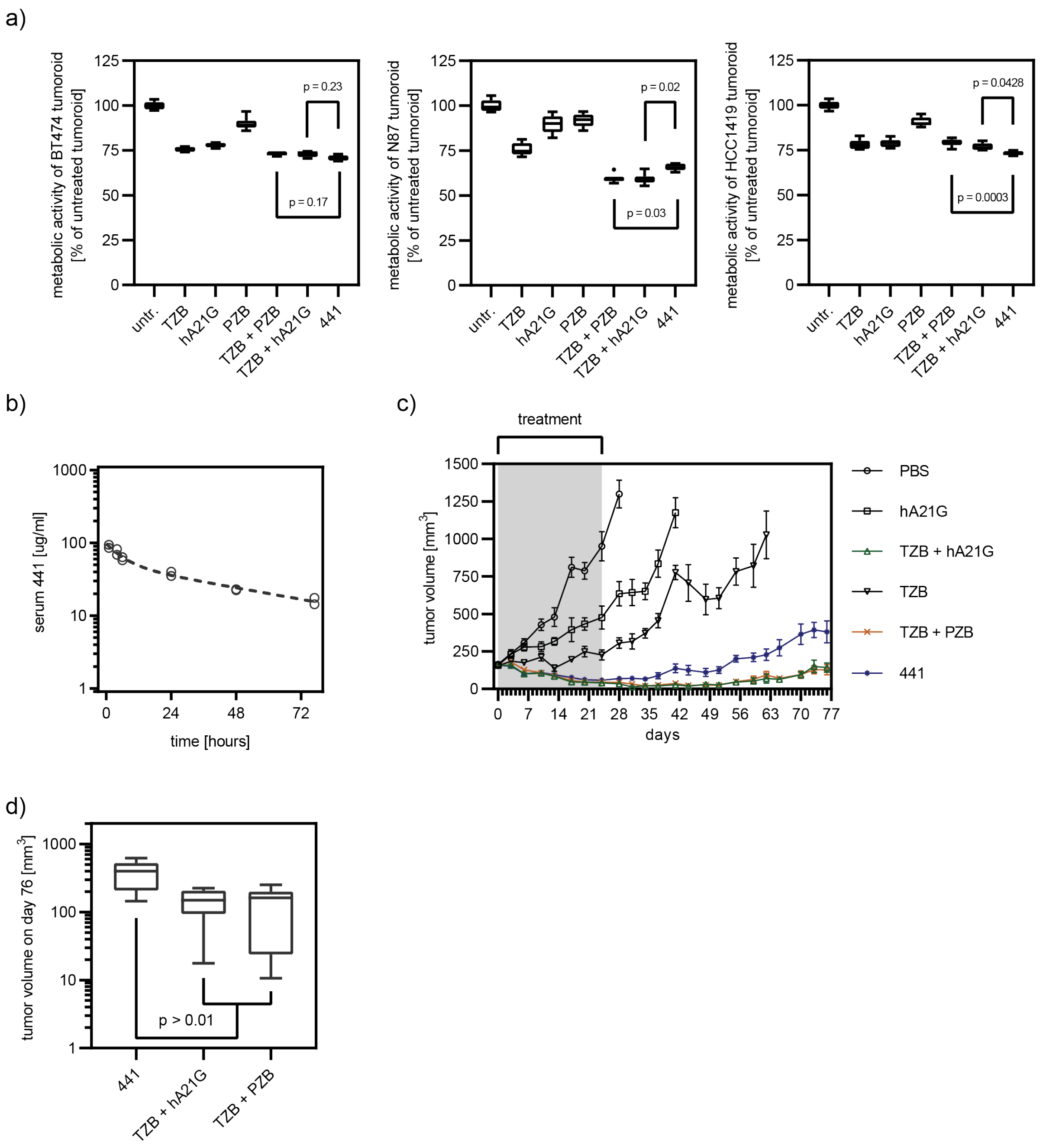

Supplementary Figure 5 – 441 remains effective in 3D spheroids models, 441 has a half-life of 45 hours and its long-term in-vivo effect was comparable to TZB combination treatments. (a) Anchorage-independent growth was equally well inhibited by 441, TZB + PZB as well as TZB + hA21G. BT474, HCC1419 and N-87 grown as spheroids were analyzed for proliferation via XTT assays (n=6, box plot with Tukey's whisker representation, ANOVA with Dunnett's multiple comparisons test) (b) The serum half-life of 441 was determined to be about 45 hours. (c) Tumor growth of N-87 xenograft model after treatment had been stopped indicating long-term responses (441 n=6, TZB n=5, hA21G n=7, TZB + PZB n=7, TZB +hA21G n=6, PBS n=7, error bar SEM). Ultimately, for none of the treatments durable effect was seen. (d) 441 was non-inferior to TZB + PZB as well as to TZB + hA21G, also on day 76 (final day of study) (441 n=6, TZB + PZB n=7, TZB +hA21G n=6, box plot with min/max whiskers, Brown-Forsythe and Welch's ANOVA with Dunnett's 3T multiple testing correction, 441 vs TZB + hA21G p=0.0385 and 441 vs TZB + PZB p=0.0324). [Source Data provided]

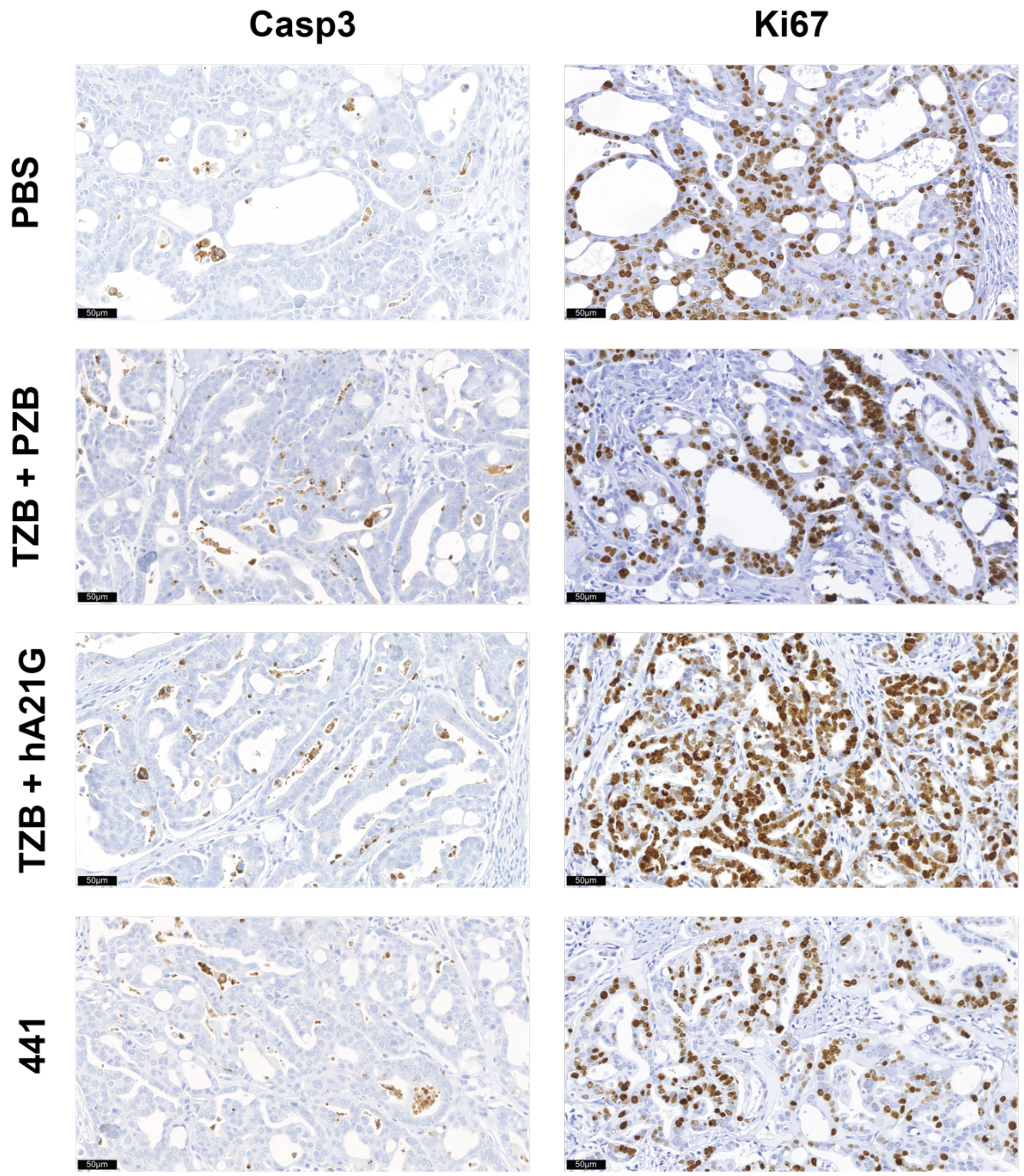

Supplementary Figure 6 – Representative IHC images of tumors under treatment. After three treatments of PBS, TZB + PZB, TZB + hA21G or 441, tumors were extracted from mice and analyzed by IHC. Tumors of mice treated with antibodies showed decreased Ki67-positive cells and about the same levels of caspase 3 positive cells.

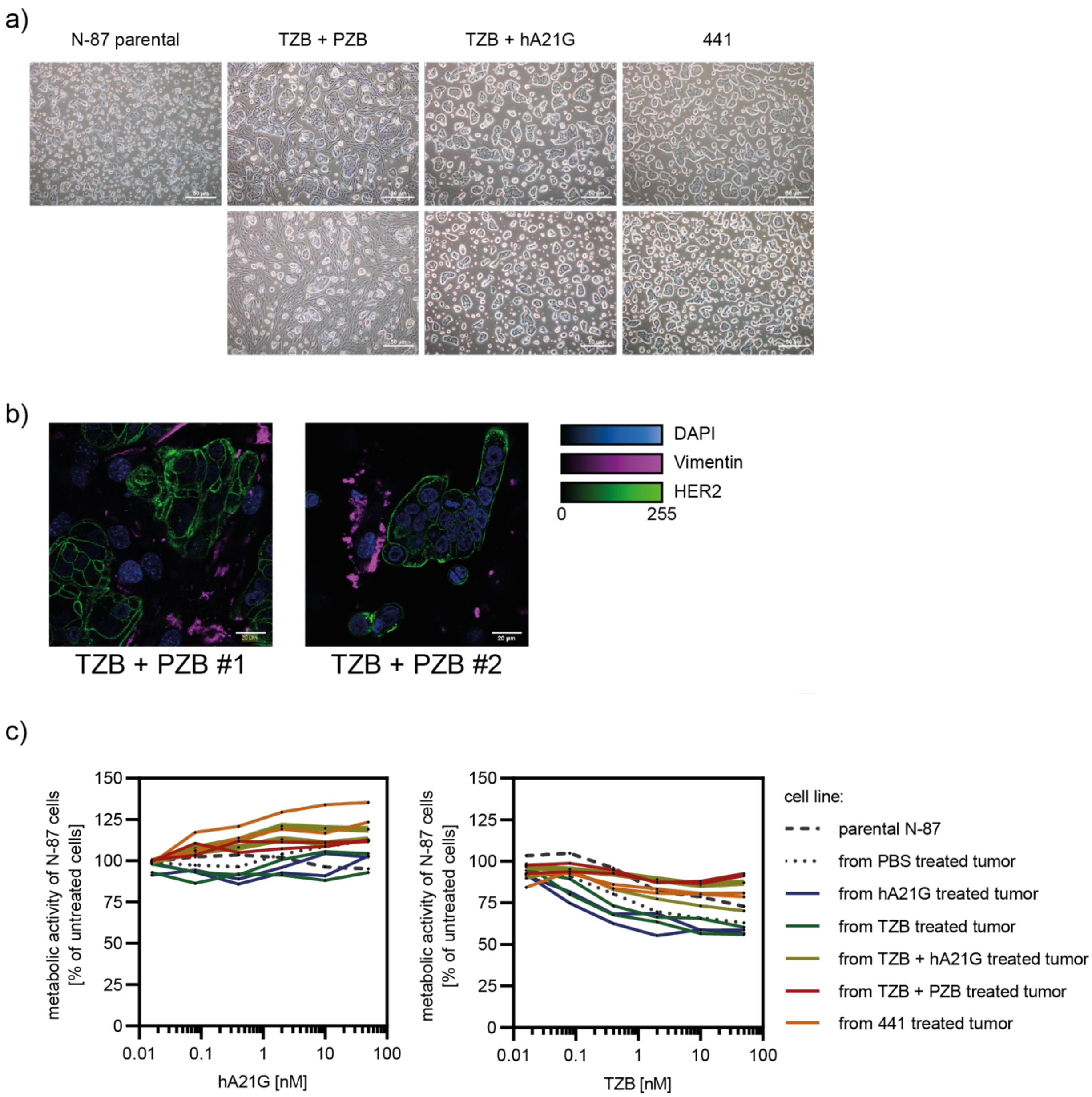

Supplementary Figure 7 – Cell lines derived from regrown tumors are partially containing fibroblasts. Cell lines are only weakly responsive to hA21G and TZB (a) Cell lines established from tumors remained morphologically similar to the N-87 parental line. For both TZB + PZB treated tumors, additionally fibroblasts were co-isolated, and prolonged cultivation did not deplete them. Pictures of passage 15 or higher are shown. (b) Fibroblasts were identified with vimentin stain, while N-87 tumor-derived cells were counterstained with a mCherry-6L1G fusion directed against HER2. (c) Cell lines derived from tumors were exposed to TZB and hA21G as single agents (n=3 biological replicates, mean plus error bar SD). Single hA21G treatment boosted proliferation in all tumor derived cell lines. TZB was only modestly effective on cell lines derived TZB or hA21G- treated tumors. The resistant cell line N-87 TZB + hA21G #2 is shown as thick olive-green line. [Source Data provided]

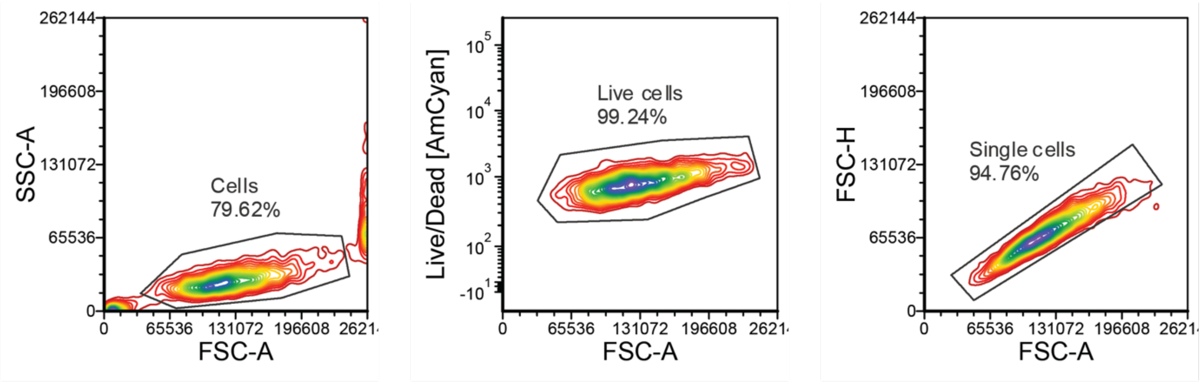

Supplementary Figure 8 – Gating strategy for flow cytometry experiments. Cells were identified from total events based on FSC-A and SSC-A. Live cells were identified from total cells using a commercial live/dead stain vs FSC-A. Single cells were identified from live cells based on FSC-H and FSC-A.

Supplementary Tables

Supplementary Table 1: Data collection and refinement statistics for structures. Values in parentheses show the data for the highest resolution shell

	841 (6ZQK)
condition	20 % PEG smear high<br>50 mM $MgCl_2$, 150 mM $LiSO_4$<br>0.1M HEPES pH 7.8
Data collection	
Resolution range	48.69 - 2.2 (2.279 - 2.2)
Space group	P1
Cell dimensions	
a, b, c (Å)	51.08, 75.64, 88.6
α, β, γ (°)	103.56, 90.33, 107.13
Total Reflections	213905 (21898)
Unique reflections	57418 (5876)
Multiplicity	3.7 (3.7)
Completeness (%)	92.07 (94.21)
I/σ(I)	10.33 (1.95)
Wilson B-factor	44.94
Rmerge	0.07633 (0.768)
Rmeas	0.0897 (0.8983)
Rpim	0.04661 (0.4624)
CC1/2	0.996 (0.584)
Refinement	
R-work	0.2028 (0.3003)
R-free	0.2401 (0.3173)
RMS(bonds)	0.004
RMS(angles)	0.76
Ramachandran plot (%)	
Favoured	96.75
Allowed	3.09
Outliers	0.15
Rotamer outliers (%)	2.47
Average B-factor (Å2)	57.9
Non-hydrogen atoms	10530
Protein	10195
Ligand	44
Water	291

Supplementary Table 2: Hydrophobic interaction chromatography runtime of constructs

name	441	TZB	hA21G	439s	239s	47C2	4MF3958	4H218
runtime [min]	7.945	5.808	7.564	7.083	7.599	6.808	8.004	5.883

Supplementary Table 3: Thermal unfolding of constructs

Name	melting temp. [°C]	95% Confidence Interval
441	68.28	68.01 to 68.55
TZB	68.94	68.70 to 69.17
hA21G	68.40	68.09 to 68.70
439s	66.26	65.95 to 66.57
239s	65.98	65.72 to 66.24
47C2	66.82	66.67 to 66.97
4MF3958	69.65	69.41 to 69.88
4H218	66.22	65.99 to 66.45

Supplementary Table 4: Commercial antibodies and used dilutions

Target	usage	dilution	Catalogue number	Supplier
HER2	WB<br>IF	1:2000<br>1:100	OP15	Merck
pHER2 (Y1221/1222)	WB<br>IF	1:2500<br>1:100	2249	Cell Signaling Technology
HER3	WB	1:1000	12708	Cell Signaling Technology
pHER3 (Y1289)	WB	1:1000	4791	Cell Signaling Technology
pAKT (S473)	WB	1:2000	4060	Cell Signaling Technology
pERK (T202/Y204)	WB	1:2000	4370	Cell Signaling Technology
PARP	WB	1:1000	9542	Cell Signaling Technology
GAPDH	WB	1:1000	sc-365062	Santa Cruz
pEGFR (Y1068)	WB	1:1000	2236	Cell Signaling Technology
EGFR	WB	1:1000	4267	Cell Signaling Technology
LAMP1	IF	1:100	15665	Cell Signaling Technology
Vimentin	IF	1:500	ab92547	abcam
Ki67	IHC	Ready to use	790-4286	Roche
Caspase 3	IHC	1:400	9664	Cell Signaling Technology
human Fc	ELISA	1:1000	209-005-098	Jackson ImmunoResearch
human Kappa-AP	ELISA	1:2000	2060-04	SouthernBiotech
human Fc AF647 labeled	IF	1:500	A-21445	Thermo Fisher Scientific
mouse IgG AF488 labeled	IF	1:500	A-11001	Thermo Fisher Scientific
anti-Rabbit IgG (H+L)	IF	1:200	O-11038	Thermo Fisher Scientific
rabbit IgG AF680 labeled	WB	1:10000	A-21076	Thermo Fisher Scientific
mouse IgG DyLight800 labeled	WB	1:10000	610-645-002	Rockland Immunochemicals Inc.

Supplementary Bibliography

1. Cheng, L. S. *et al.* Construction, expression and characterization of the engineered antibody against tumor surface antigen, p185c-erbB-2. *Cell Res.* **13**, 35–48 (2003).

2. Tamaskovic, R., Simon, M., Stefan, N., Schwill, M. & Plückthun, A. Designed ankyrin repeat proteins (DARPins). *Methods Enzymol.* **503**, 101–134 (2012).

3. Hacker, D. L. *et al.* Polyethyleneimine-based transient gene expression processes for suspension-adapted HEK-293E and CHO-DG44 cells. *Protein Expr. Purif.* **92**, 67–76 (2013).

4. Kabsch, W. XDS. *Acta Crystallogr. Sect. D Biol. Crystallogr.* **66**, 125–132 (2010).

5. McCoy, A. J. *et al.* Phaser crystallographic software. *J. Appl. Crystallogr.* **40**, 658–674 (2007).

6. Emsley, P., Lohkamp, B., Scott, W. G. & Cowtan, K. Features and development of Coot. *Acta Crystallogr. Sect. D Biol. Crystallogr.* **66**, 486–501 (2010).

7. Murshudov, G. N. *et al.* REFMAC 5 for the refinement of macromolecular crystal structures. *Acta Crystallogr. Sect. D Biol. Crystallogr.* **67**, 355–367 (2011).

8. Afonine, P. V. *et al.* Towards automated crystallographic structure refinement with phenix.refine. *Acta Crystallogr. Sect. D Biol. Crystallogr.* **68**, 352–367 (2012).

9. Bricogne, G. *et al.* BUSTER version 2.10.3. *Cambridge, United Kingdom Glob. Phasing Ltd.* (2017).

10. Joosten, R. P., Joosten, K., Murshudov, G. N. & Perrakis, A. PDB_REDO : constructive validation, more than just looking for errors. *Acta Crystallogr. Sect. D Biol. Crystallogr.* **68**, 484–496 (2012).

11. Karplus, P. A. & Diederichs, K. Linking crystallographic model and data quality. *Science* **336**, 1030–3 (2012).

12. Tamaskovic, R. *et al.* Intermolecular biparatopic trapping of ErbB2 prevents compensatory activation of PI3K/AKT via RAS–p110 crosstalk. *Nat. Commun.* **7**, 11672 (2016).
