# Supplementary material for: Engineering an anti-HER2 biparatopic antibody with a multimodal mechanism of action
Source: Nat Commun. 2021 Jun 18;12:3790. doi: 10.1038/s41467-021-23948-6 (PMC8213836; doi:10.1038/s41467-021-23948-6)
Supplement: Supplementary file 2 — Reporting Summary [file 41467_2021_23948_MOESM2_ESM.pdf]

nature research

Corresponding author(s): Prof. Andreas Plückthun

Last updated by author(s): Jan 31, 2021

Reporting Summary

Nature Research wishes to improve the reproducibility of the work that we publish. This form provides structure for consistency and transparency in reporting. For further information on Nature Research policies, see our Editorial Policies and the Editorial Policy Checklist.

Statistics

For all statistical analyses, confirm that the following items are present in the figure legend, table legend, main text, or Methods section.

n/a	Confirmed	
☐	☒	The exact sample size (*n*) for each experimental group/condition, given as a discrete number and unit of measurement
☐	☒	A statement on whether measurements were taken from distinct samples or whether the same sample was measured repeatedly
☐	☒	The statistical test(s) used AND whether they are one- or two-sided<br>*Only common tests should be described solely by name; describe more complex techniques in the Methods section.*
☒	☐	A description of all covariates tested
☐	☒	A description of any assumptions or corrections, such as tests of normality and adjustment for multiple comparisons
☐	☒	A full description of the statistical parameters including central tendency (e.g. means) or other basic estimates (e.g. regression coefficient) AND variation (e.g. standard deviation) or associated estimates of uncertainty (e.g. confidence intervals)
☐	☒	For null hypothesis testing, the test statistic (e.g. *F*, *t*, *r*) with confidence intervals, effect sizes, degrees of freedom and *P* value noted<br>*Give P values as exact values whenever suitable.*
☒	☐	For Bayesian analysis, information on the choice of priors and Markov chain Monte Carlo settings
☒	☐	For hierarchical and complex designs, identification of the appropriate level for tests and full reporting of outcomes
☐	☒	Estimates of effect sizes (e.g. Cohen's *d*, Pearson's *r*), indicating how they were calculated

Our web collection on statistics for biologists contains articles on many of the points above.

Software and code

Policy information about availability of computer code

Data collection	Gen5 V3.05+, Image Studio V5+, ASTRA V6.1+, FACS Diva V8,
Data analysis	GraphPad Prism V8+, Image Study Lite V5.2+, Fiji, switchANALYSIS V1.9+, FlowJo V10, FCS Express V5+, Trimmomatic V0.36 , STAR aligner V2.5.2b, RStudio V1.3+, Visiopharm V5.0.4. 1382, CellProfiler V2.2.0, XDS, XSCALE, XDSCONV, PHASER, Coot, refMAC5, PHENIX refine, BUSTER, pdb_redo, pymol V2.4+

For manuscripts utilizing custom algorithms or software that are central to the research but not yet described in published literature, software must be made available to editors and reviewers. We strongly encourage code deposition in a community repository (e.g. GitHub). See the Nature Research guidelines for submitting code & software for further information.

Data

Policy information about availability of data

All manuscripts must include a data availability statement. This statement should provide the following information, where applicable:

- Accession codes, unique identifiers, or web links for publicly available datasets
- A list of figures that have associated raw data
- A description of any restrictions on data availability

Coordinates and structure factors of 841 were deposited in the Protein Data Bank under Accession Code [6ZQK] with corresponding crystallographic data collection and refinement statistics shown in Supplementary Table 1. All other data is attached and in the source file.

Field-specific reporting

Please select the one below that is the best fit for your research. If you are not sure, read the appropriate sections before making your selection.

- [x] Life sciences
- [ ] Behavioural & social sciences
- [ ] Ecological, evolutionary & environmental sciences

For a reference copy of the document with all sections, see nature.com/documents/nr-reporting-summary-flat.pdf

Life sciences study design

All studies must disclose on these points even when the disclosure is negative.

Sample size	Sample size for in vivo experiments was calculated. Deduced from preliminary efficacy seen in a pilot study and literature the effect size was estimated and the resulting sample size was calculated with alpha = 0.05 and a power of 0.80.<br>For in vitro experiments no sample size calculations were done beforehand. Estimates were based on ref 7, 14, 27-28, and 50.
Data exclusions	No recorded data was excluded from analysis
Replication	All experiments were repeated successfully at least once with the exact same settings. The in vivo study and the crystallization were not repeated.
Randomization	In the in vivo study animals were grouped based on their initial tumor burden to have an equal starting point. Animals were assigned to the group by one author, groups were allocated to treatments by another author not involved in the in vivo part of the study. Animals stayed in their original cages and where not moved to form cages only having one single cohort. For in vitro experiments, samples were allocated to predefined wells (pipetting scheme) and we think the spatial location of the sample/well influences experimental results.
Blinding	All researchers involved in the in-vivo study were blinded during the experiment for the assignment of the mice to cohorts, and tumor growth data was for the first time analyzed after all injections had been performed. For in-vitro studies experiments were performed by a single person, thus blinding was impracticable.

Reporting for specific materials, systems and methods

We require information from authors about some types of materials, experimental systems and methods used in many studies. Here, indicate whether each material, system or method listed is relevant to your study. If you are not sure if a list item applies to your research, read the appropriate section before selecting a response.

Materials & experimental systems

n/a	Involved in the study	
	x	Antibodies
	x	Eukaryotic cell lines
x		Palaeontology and archaeology
	x	Animals and other organisms
x		Human research participants
x		Clinical data
x		Dual use research of concern

Methods

n/a	Involved in the study	
x		ChIP-seq
	x	Flow cytometry
x		MRI-based neuroimaging

Antibodies

Antibodies used	All commercial antibodies are listed Supplementary Table 4 including for which method they were used for in which dilution.
Validation	We did not validate antibodies. Commercial antibodies were only used for the designated species and validated use provided by the manufacture.<br>All CST antibodies: https://www.cellsignal.com/about-us/our-approach-process/cst-antibody-validation-principles<br>All ThermoFischer: https://www.thermofisher.com/ch/en/home/life-science/antibodies/invitrogen-antibody-validation.html<br>Santa Cruz: GAPDH antibody cited in 1,000+ publications<br>Merck: https://www.sigmaaldrich.com/life-science/cell-biology/antibodies/antibody-validation.html<br>abcasm: Our Abpromise guarantee covers the use of ab92547 in the following tested applications: IF<br>Ki67 is an antibody that is accredited (Norm ISO/IEC 17025)<br>Jackson ImmunoResearch: human Fc - Based on immunoelectrophoresis and/or ELISA, the antibody reacts with the Fc portion of human IgG heavy chain but not with the Fab portion of human IgG. No antibody was detected against human IgM or IgA, or against non-immunoglobulin serum proteins. The antibody exhibits inherent minimal cross-reaction to mouse serum proteins and has been tested by ELISA and/or solid-phase adsorbed to ensure minimal cross-reaction with bovine, horse, and mouse serum proteins. The antibody may cross-react with immunoglobulins from other species.

Southern Biotech: human kappa-AP - Quality tested applications include ELISA

Eukaryotic cell lines

Policy information about cell lines

Cell line source(s)	The following cell lines were purchased from ATCC: AU565, BT474, Calu-3, HCC1419, HCC2218, H358, MCF7, N-87, SKBR3, UACC-893, and ZR-75-30. Cell lines CW-2 and MKN-7 were obtained from RIKEN Biobank, Japan. Hela cells for the single molecule tracking experiments were provided by the laboratory of Prof. J. Piehler.
Authentication	Cell lines were not further authenticated by us.
Mycoplasma contamination	All cell lines routinely tested negative for mycoplasma contamination.
Commonly misidentified lines (See ICLAC register)	MKN-7 were obtained from RIKEN Biobank, Japan. RIKEN Biobank is the authentic source of this commonly misidentified cell line. We are confident we used the right cell line.

Animals and other organisms

Policy information about studies involving animals; ARRIVE guidelines recommended for reporting animal research

Laboratory animals	female NSG mice (NOD.Cg-Prkdcscid Il2rgtm1WjI/SzJ; Charles River) at least 4 weeks old<br>female Fox Chase SCID Beige mice (CB17.Cg-PrkdcscidLystbg-J/Crl; Charles River) at least 4 weeks old
Wild animals	no wild animals studied
Field-collected samples	no field-collected samples were studied
Ethics oversight	All mice experiments were performed in accordance with the Swiss animal protection law and with approval of the Cantonal Veterinary Office (Zurich, Switzerland).<br>Additional guidance was provided by the Zurich Integrative Rodent Physiology (ZIRP) team. ZIRP is an interdisciplinary core facility within the University of Zurich .

Note that full information on the approval of the study protocol must also be provided in the manuscript.

Flow Cytometry

Plots

Confirm that:

- [ ] The axis labels state the marker and fluorochrome used (e.g. CD4-FITC).
- [ ] The axis scales are clearly visible. Include numbers along axes only for bottom left plot of group (a 'group' is an analysis of identical markers).
- [ ] All plots are contour plots with outliers or pseudocolor plots.
- [ ] A numerical value for number of cells or percentage (with statistics) is provided.

Methodology

Sample preparation	Cell lines were detached by the use of trypsin, detached cells were washed twice with PBS. Stained as described in material and methods. Filtered by the use of tubes with cell strainer snap caps.
Instrument	BD LSR II Fortessa with HTS and FACS Canto II with HTS maintained by the Cytometry Facility of the University of Zurich
Software	FACS Diva V8+ for collection and FlowJo V10 or FCS Express V5 for analysis
Cell population abundance	Only cell lines where analyzed by flow cytometry. We analyzed the viable single cell subpopulation which was normally >90%. Two cell lines derived from mouse tumor had around 10% fibroblasts contamination within the tumor cell line. Their presence was confirmed by microscopy and this is described in our manuscript.
Gating strategy	Cells were gated for viable cells (commercial live/dead stain) and gated for single cells by FCS-A vs FCS-H

- [ ] Tick this box to confirm that a figure exemplifying the gating strategy is provided in the Supplementary Information.
