# Supplementary figures and images for: Engineering an anti-HER2 biparatopic antibody with a multimodal mechanism of action
Source: Nat Commun. 2021 Jun 18;12:3790. doi: 10.1038/s41467-021-23948-6 (PMC8213836; doi:10.1038/s41467-021-23948-6)

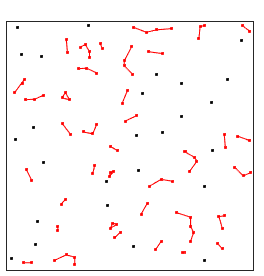

Supplement: Supplementary file 3 — Supplementary Data 1 [file 41467_2021_23948_MOESM3_ESM.zip › Release/96mer_link=18nm_d=100nm_surfaceSideLength=1000nm_2_sites/Surface10Simulation100.png]

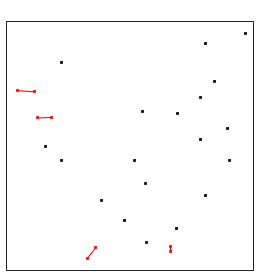

Supplement: Supplementary file 3 — Supplementary Data 1 [file 41467_2021_23948_MOESM3_ESM.zip › Release/96mer_link=18nm_d=200nm_surfaceSideLength=1000nm_2_sites/Surface19Simulation1000.png]

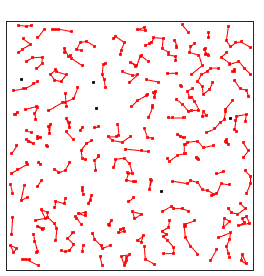

Supplement: Supplementary file 3 — Supplementary Data 1 [file 41467_2021_23948_MOESM3_ESM.zip › Release/96mer_link=18nm_d=50nm_surfaceSideLength=1000nm_2_sites/Surface10Simulation100.png]

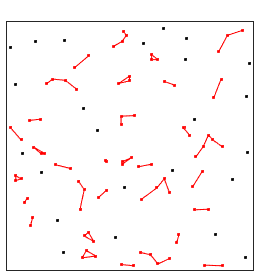

Supplement: Supplementary file 3 — Supplementary Data 1 [file 41467_2021_23948_MOESM3_ESM.zip › Release/96mer_link=30nm_d=100nm_surfaceSideLength=1000nm_2_sites/Surface10Simulation100.png]

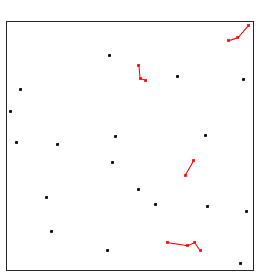

Supplement: Supplementary file 3 — Supplementary Data 1 [file 41467_2021_23948_MOESM3_ESM.zip › Release/96mer_link=30nm_d=200nm_surfaceSideLength=1000nm_2_sites/Surface30Simulation2100.png]

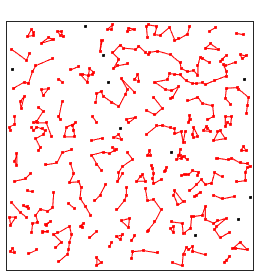

Supplement: Supplementary file 3 — Supplementary Data 1 [file 41467_2021_23948_MOESM3_ESM.zip › Release/96mer_link=30nm_d=50nm_surfaceSideLength=1000nm_2_sites/Surface10Simulation100.png]

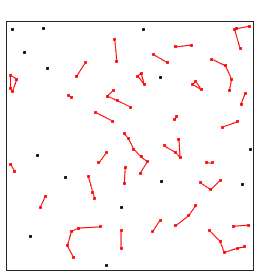

Supplement: Supplementary file 3 — Supplementary Data 1 [file 41467_2021_23948_MOESM3_ESM.zip › Release/96mer_link=36nm_d=100nm_surfaceSideLength=1000nm_2_sites/Surface10Simulation100.png]

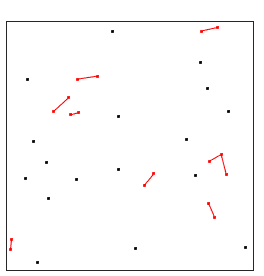

Supplement: Supplementary file 3 — Supplementary Data 1 [file 41467_2021_23948_MOESM3_ESM.zip › Release/96mer_link=36nm_d=200nm_surfaceSideLength=1000nm_2_sites/Surface17Simulation800.png]

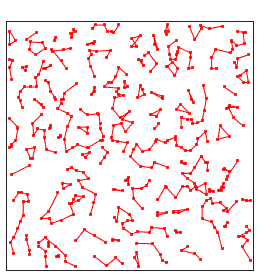

Supplement: Supplementary file 3 — Supplementary Data 1 [file 41467_2021_23948_MOESM3_ESM.zip › Release/96mer_link=36nm_d=50nm_surfaceSideLength=1000nm_2_sites/Surface10Simulation100.png]
