# Supplementary material for: Engineering an anti-HER2 biparatopic antibody with a multimodal mechanism of action
Source: Nat Commun. 2021 Jun 18;12:3790. doi: 10.1038/s41467-021-23948-6 (PMC8213836; doi:10.1038/s41467-021-23948-6)
Supplement: Supplementary file 4 — Description of Additional Supplementary Files [file 41467_2021_23948_MOESM4_ESM.pdf]

Title: Supplementary Data 1

Description: Binding simulations of nanolever crosslinking events (defined as at least two nanolevers being interlinked by the analyte) to HER2 on DNA nanolevers using the switchSENSE technology. The pictures show examples of simulated surfaces of 1 × 1 µm, depicting interlinking of HER2-DNA conjugates. The simulations were run for different densities (50, 100, 200 nm average distance between nanolevers) and different crosslinker sizes (441 (ca. 20 nm), 841 (ca. 2 nm), and TZB and hA21G IgGs (ca. 14 nm). Corresponding MATLAB files for the simulations on each of the 10 surfaces are included.
